# Bayesian variable selection in linear quantile mixed models for longitudinal data with application to macular degeneration

**DOI:** 10.1371/journal.pone.0241197

**Published:** 2020-10-26

**Authors:** Yonggang Ji, Haifang Shi

**Affiliations:** School of Science, Civil Aviation University of China, Tianjin, China; Yunnan University of Finance and Economics, CHINA

## Abstract

This paper presents a Bayesian analysis of linear mixed models for quantile regression based on a Cholesky decomposition for the covariance matrix of random effects. We develop a Bayesian shrinkage approach to quantile mixed regression models using a Bayesian adaptive lasso and an extended Bayesian adaptive group lasso. We also consider variable selection procedures for both fixed and random effects in a linear quantile mixed model via the Bayesian adaptive lasso and extended Bayesian adaptive group lasso with spike and slab priors. To improve mixing of the Markov chains, a simple and efficient partially collapsed Gibbs sampling algorithm is developed for posterior inference. Simulation experiments and an application to the Age-Related Macular Degeneration Trial data to demonstrate the proposed methods.

## Introduction

Quantile regression(QR) in longitudinal or panel data models have received increasing attention in recent years. For example, [1] proposed a general method to estimate the QR coefficients by using a *L*_1_ penalized likelihood approach. Later [2] generalized the work of [1] to be more flexible model with endogenous variables. [3] offered a estimation of individual effects for panel data QR models, which is widely applied by practitioners because of computationally simple. [4] studied quantile panel models with correlated random effects. [5] proposed a Stochastic Approximation of the EM (SAEM) algorithm to analyze linear quantile mixed regressions(LQMMs) via the asymmetric Laplace distribution. Other related papers include [6–9], among many others.

Like all regression issues, when there are many covariates in longitudinal or panel QR models, variable selection becomes necessary to avoid overfitting and multicollinearity. [10] presented a penalized quantile regression model for random intercept using the Bayesian lasso priors and Bayesian adaptive lasso priors, respectivly. [11] also considered a Bayesian Lasso approach to jointly estimate a vector of covariate effects and a vector of random effects by introducing an *l*_1_ penalty. However, for the random effects, their method mainly focus on the penalty of diagonal elements in the covariance matrix of random effects after a modified Cholesky decomposition. In addition, they neglect the nonnegative constraint on the diagonal terms in the covariance matrix of random effects after a modified Cholesky decomposition, which can cause non-uniqueness in the decomposition [12]. Then, we first propose a new Bayesian shrinkage approach to solve these problems in the LQMMs. In particular, we develop an extended Bayesian adaptive group lasso which can accommodate the nonnegative constraint of the diagonal terms to attempt to identify the non-zero random effects. Unfortunately, the shrinkage approach need some ad hoc methods to do variable selection similar to [10] and [11] for the well known reason. An alternative to the Bayesian variable selection is to use spike and slab priors. We then develop a general variable selection technic via the Bayesian adaptive lasso and the extended Bayesian adaptive group lasso with spike and slab priors to identify the significant fixed and random effects simultaneously.

The paper is organized as follows. Section 2 presents the reparameterized LQMMs based on a Cholesky decomposition of covariance matrix of random effects. Section 3 describes the structure of our hierarchical Bayesian LQMMs and discuss our prior specifications. We also develop a Partially collapsed Gibbs (PCG) sampling algorithm for posterior inference. In section 4, we will develop a variable selection procedure for both fixed and random effects in a LQMM with spike and slab priors and an efficient PCG. We illustrate the performance of our method by simulation studies and a real data example in section 5 and 6. Results show that the proposed approach performs very well. In Section 7, we conclude the paper.

## The re-parameterized LQMMs

Consider *n* subjects containing *n*_*i*_ observations, $\left(y_{ij},x_{ij}^{\prime },z_{ij}^{\prime }\right)$, for *j* = 1, …, *n*_*i*_ and $i=1,\ldots ,n,N=\sum_{i=1}^{n}n_{i}$, where *y*_*ij*_ is the *j*th response for subject *i*, $x_{ij}^{\prime }$ is the *j*th row of a known *n*_*i*_ × *p* matrix ***x**_i_* and $z_{ij}^{\prime }$ is the *j*th row of a known *n*_*i*_ × *q* matrix ***z**_i_*. The canonical linear mixed model is as follows:

$$\begin{array}{c} y_{ij}=x_{ij}^{\prime }\beta +z_{ij}^{\prime }\alpha_{i}+\varepsilon_{ij},\alpha_{i}∼N\left(0,D\right), \end{array}$$ (1)

where ***β*** is a *p* × 1 vector of unknown population fixed parameters, ***α**_i_* is a *q* × 1 vector of unobservable random effects with covariance matrix ***D***. In order to guarantee the positive semidefinite of ***D***, we adopt the Cholesky decomposition to it, resulting in

$$D={\Gamma \Gamma }^{T},$$

where **Γ** = (*γ_st_*) is a *q* × *q* lower triangular matrix with nonnegative diagonal entries. Similar to [13], the model (1) can be re-expressed as follows:

$$\begin{array}{c} y_{ij}=x_{ij}^{\prime }\beta +z_{ij}^{\prime }\Gamma b_{i}+\varepsilon_{ij}=x_{ij}^{\prime }\beta +\left(b_{i}^{T}⊗z_{ij}^{\prime }\right)J_{q}\gamma +\varepsilon_{ij},b_{i}∼N\left(0,I_{q}\right). \end{array}$$ (2)

Here $\gamma^{T}=\left(\gamma_{1}^{T},\ldots ,\gamma_{q}^{T}\right)$ is a 1 × *q*(*q* + 1)/2 vector, where *γ_k_* consists of the non-zero lower triangular elements of the kth row of **Γ**, i.e., $\gamma_{k}={\left(\gamma_{k1},\ldots ,\gamma_{kk}\right)}^{T}$, *k* = 1, …, *q*, ***J**_q_* is a transformation matrix with size *q*^2^ × *q*(*q* + 1)/2 such that *vec*(**Γ**) = ***J**_q_*γ, **I**_*q*_ denotes the *q* × *q* identity matrix. For example, if *q* = 2

$$\begin{array}{c} \Gamma =(\begin{array}{cc} \gamma_{11} & 0\\ \gamma_{21} & \gamma_{22} \end{array}), \end{array}$$

the transformation matrix ***J***_2_ would have the following form

$$\begin{array}{c} J_{2}=(\begin{array}{ccc} 1 & 0 & 0\\ 0 & 1 & 0\\ 0 & 0 & 0\\ 0 & 0 & 1 \end{array}). \end{array}$$

With model (2), the linear conditional *τ*th (0 < *τ* < 1) mixed quantile estimator can be calculated by

$$\begin{array}{c} \underset{\beta ,\gamma }{\text{min}}\sum_{i=1}^{n}\sum_{j=1}^{n_{i}}\rho_{\tau }\left(y_{ij}-x_{ij}^{\prime }\beta -\left(b_{i}^{T}⊗z_{ij}^{\prime }\right)J_{q}\gamma \right). \end{array}$$ (3)

In a Bayesian setup, this is equivalent to assuming that the error terms *ε*_*ij*_ in (2) follow the asymmetric Laplace distribution (ALD), of which the probability density function is given by

$$\begin{array}{c} f_{p}\left(\varepsilon_{ij}\right)=\tau \left(1-\tau \right)\sigma \;\text{exp}\left\{-\sigma \rho_{\tau }\left(\varepsilon_{ij}\right)\right\},\rho_{\tau }\left(\varepsilon_{ij}\right)=\varepsilon_{ij}\left\{\tau -I\left(\varepsilon_{ij}<0\right)\right\}, \end{array}$$ (4)

where *σ*^−1^ and *τ* are the scale and skewness parameters, respectively. [14] first used the ALD to establish Bayesian QR in a linear model for independent data and proposed a random walk Metropolis-Hastings algorithm to draw samples. Since then, there has been a great deal of literature on the extension and application of the QR based on the ALD such as [15–18]. However, direct sampling using the ALD is often inconvenient and not easy to generalize to more complex scenarios. We employ a mixture representation of ALD proposed by [19] to facilitate Bayesian inference in LQMMs, which was also used by many authors (e.g., see [11, 20–22]),

$$\begin{array}{c} y_{ij}=x_{ij}^{\prime }\beta +z_{ij}^{\prime }\Gamma b_{i}+\xi_{1}v_{ij}+\xi_{2}\sigma^{-1/2}{\sqrt{v}}_{ij}\zeta_{ij},j=1,\ldots ,n_{i},i=1,\ldots ,n, \end{array}$$ (5)

where *v*_*ij*_ ∼ *exp*(1/*σ*) has an exponential prior with mean 1/*σ*, *ζ*_*ij*_ ∼ *N*(0, 1) has a standard normal prior and is independent of *v*_*ij*_, $\xi_{1}=\frac{1-2\tau }{\tau (1-\tau )}$ and $\xi_{2}^{2}=\frac{2}{\tau (1-\tau )}$. Let ***v*** = (*v_ij_*: *i* = 1, …, *n*; *j* = 1, …, *n_i_*), ***ζ*** = (*ζ_ij_*: *i* = 1, …, *n*; *j* = 1, …, *n_i_*). Hence, based on the Cholesky decomposition and the mixture representation of ALD, we obtain the following hierarchal model,

$$\begin{array}{ccc} y_{ij} & = & x_{ij}^{\prime }\beta +\left(b_{i}^{T}⊗z_{ij}^{\prime }\right)J_{q}\gamma +\xi_{1}v_{ij}+\xi_{2}\sigma^{-1/2}{\sqrt{v}}_{ij}\zeta_{ij},\\ v|\sigma & ∼ & \prod_{i=1}^{n}\prod_{j=1}^{n_{i}}\sigma \;\text{exp}\left(-\sigma v_{ij}\right),\\ \zeta & ∼ & \prod_{i=1}^{n}\prod_{j=1}^{n_{i}}\frac{1}{\sqrt{2\pi }}\text{exp}\left(-\frac{1}{2}\zeta_{ij}^{2}\right). \end{array}$$

## Shrinkage estimation of linear quantile mixed regression

We first propose a new Bayesian shrinkage approach in LQMMs by using the Bayesian adaptive lasso and an extended Bayesian adaptive group lasso. A regularized *τ*th quantile estimates of the fixed and random effect coefficients can be defined by

$$\begin{array}{c} \begin{array}{c} \text{arg}\;\underset{\beta ,\gamma }{\text{min}}\sum_{i=1}^{n}\sum_{j=1}^{n_{i}}\rho_{\tau }\left(y_{ij}-x_{ij}^{\prime }\beta -\left(b_{i}^{T}⊗z_{ij}^{\prime }\right)J_{q}\gamma \right)+\sum_{s=1}^{p}\lambda_{1s}^{\prime }\left|\beta_{s}\right|+\sum_{k=1}^{q}\lambda_{2k}^{\prime }{\left(\gamma_{k}^{T}\gamma_{k}\right)}^{1/2}\\ \;\;\;\;\;\;s.t.\;\gamma_{kk}\geq 0,k=1,...q. \end{array} \end{array}$$ (6)

Motivated by [23], we put a Laplace priors $\pi \left(\beta_{s}|\sigma ,\lambda_{1s}^{\prime }\right)=C_{1}exp\{-\sigma \sum \lambda_{1s}^{\prime }|\beta_{s}\left|\right\}$ on *β*_*s*_, where $C_{1}=\sigma \lambda_{1s}^{\prime }/2$, a Laplace priors $\pi \left(\gamma_{k}|\sigma ,\lambda_{2k}^{\prime }\right)=C_{2}exp\left\{-\sigma \lambda_{2k}^{\prime }{\left(\gamma_{k}^{T}\gamma_{k}\right)}^{1/2}\right\}$, where $C_{2}=2^{-(k+1)/2}{\left(2\pi \right)}^{-(k-1)/2}{\left(\sigma \lambda_{2k}^{\prime }\right)}^{k}/\Gamma \left(\left(k+1\right)/2\right)$, and suppose that the error terms *ε*_*ij*_ come from the ALD (4). Then, the posterior distribution of ***β*** and ***γ*** is proportional to

$$\begin{array}{c} \begin{array}{c} exp\{-\sigma \rho_{\tau }\left(y_{ij}-x_{ij}^{\prime }\beta -\left(b_{i}^{T}⊗z_{ij}^{\prime }\right)J_{q}\gamma \right)-\sigma \sum_{s=1}^{p}\lambda_{1s}^{\prime }|\beta_{s}|-\sigma \sum_{k=1}^{q}\lambda_{2k}^{\prime }{\left(\gamma_{k}^{T}\gamma_{k}\right)}^{1/2}\}. \end{array} \end{array}$$ (7)

So it can be easily observed that maximizing the posterior distribution (7) is equivalent to minimizing the target function (6) if we ignore the nonnegative constraints *γ*_*kk*_ ≥ 0, *k* = 1, …*q*. However, it is not clear how to incorporate this constraints within the Bayesian Penalized LQMMs framework. Using truncated normal priors, [24] and [25] solved a similar problem on the selection of fixed effects, which is non-group structure in linear and generalized mixed model, respectively. We extend the idea to the Bayesian Group lasso on the shrinkage of random effects with group structure. We specify the conditional prior distributions of ***γ**_k_* as $\pi \left(\gamma_{k}|\sigma ,\left\{\lambda_{2k}^{\prime }\right\}\right)=2C_{2}exp\left\{-\sigma \lambda_{2k}^{\prime }{\left(\gamma_{k}^{T}\gamma_{k}\right)}^{1/2}\right\}I\left(\gamma_{kk}\geq 0\right)$. To facilitate posterior inference, we utilize a mixture representation to yield

$$\begin{array}{ccc} \pi (\gamma_{k}|\lambda_{2k}) & = & 2C_{2}exp\left\{-\lambda_{2k}{\left(\gamma_{k}^{T}\gamma_{k}\right)}^{1/2}\right\}I\left(\gamma_{kk}\geq 0\right)\\ & = & \int_{0}^{+\infty }2{\left(\frac{1}{\sqrt{2\pi \eta_{k}}}\right)}^{k}\text{exp}(-\frac{\gamma_{k}^{\prime }\gamma_{k}}{2\eta_{k}})I\left(\gamma_{kk}\geq 0\right)\\ & \times & \frac{{\left(\lambda_{2k}^{2}/2\right)}^{(k+1)/2}}{\Gamma ((k+1)/2)}\eta_{k}^{\frac{\left(k+1\right)}{2}-1}exp(-\frac{\lambda_{2k}^{2}}{2}\eta_{k})\;d\eta_{k}, \end{array}$$

where $\lambda_{2k}=\sigma \lambda_{2k}^{\prime }$. Then, ***γ**_k_* can be expressed hierarchically as

$$\begin{array}{c} \gamma_{k}\left|\eta_{k}∼N_{k}\left(0,\eta_{k}I_{k}\right)\;\;\;\;\;\;\;\;and\;\;\;\;\;\;\;\;\eta_{k}\right|\lambda_{2k}^{2}∼Gamma(\frac{k+1}{2},\frac{\lambda_{2k}^{2}}{2}), \end{array}$$ (8)

for *k* = 1, …, *q*. Moving to the fixed effects parameters, ***β*** can also be written hierarchically as follows

$$\begin{array}{c} \beta_{s}\left|t_{s}∼N\left(0,t_{s}\right)\;\;\;\;\;\;\;\;and\;\;\;\;\;\;\;\;t_{s}\right|\lambda_{1s}^{2}∼Gamma(1,\frac{\lambda_{1s}^{2}}{2}), \end{array}$$ (9)

for *s* = 1, …, *p*. There are two main ways to estimate the tuning parameter in Bayesian regularization: first, a fully Bayesian method, which specifies a hyperprior on it, such as a conjugate Gamma distribution [23], and second, an empirical Bayesian method, which estimates it using maximum marginal likelihood method [26]. Here we use the fully Bayesian approach and set Gamma priors on the parameters $\lambda_{1s}^{2}$, $\lambda_{2k}^{2}$ and *σ* to complete the Bayesian structure. We develop a Partially collapsed Gibbs (PCG) sampling sampler by integrating the full conditional distribution of ***β*** with respect to {*b*_*j*_} to sample from posterior distributions. In order to improve the convergence behavior of the Gibbs sampler, [27] proposed PCG which is a generalization of blocking and collapsing method [28]. The key idea of the PCG is to replace some of the posterior distributions with marginal posterior distribution while preserving the target distribution. Next, we present the conditional distribution of unknown parameters.

(1) Conditional distribution of ***β***:

Let ***T*** = *diag*(*t_s_*, *s* = 1, …, *p*) and $w_{ij}=\left(z_{ij}^{\prime }\Gamma \right){\left(z_{ij}^{\prime }\Gamma \right)}^{\prime }+\xi_{2}^{2}\sigma^{-1}v_{ij}$. The conditional distribution of ***β*** is then a $N({\hat{\mu }}_{\beta },{\hat{\Sigma }}_{\beta })$, where

$$\begin{array}{c} {\hat{\Sigma }}_{\beta }={\left[\sum_{i=1}^{n}\sum_{j=1}^{n_{i}}x_{ij}w_{ij}x_{ij}^{\prime }+T^{-1}\right]}^{-1},\;\;{\hat{\mu }}_{\beta }={\hat{\Sigma }}_{\beta }\sum_{i=1}^{n}\sum_{j=1}^{n_{j}}x_{ij}w_{ij}\left(y_{ij}-\xi_{1}v_{ij}\right). \end{array}$$

(2) Conditional distribution of ***b**_i_*:

The conditional distribution of ***b**_i_* is a multivariate normal distribution with mean ${\hat{\mu }}_{b_{i}}$ and variance ${\hat{\Sigma }}_{b_{i}}$ where

$$\begin{array}{c} {\hat{\Sigma }}_{b_{i}}={\left[\sum_{j=1}^{n_{i}}\frac{{\left(z_{ij}^{\prime }\Gamma \right)}^{\prime }\left(z_{ij}^{\prime }\Gamma \right)}{2\sigma^{-1}v_{ij}}+I_{q}\right]}^{-1},\;\;{\hat{\mu }}_{b_{i}}={\hat{\Sigma }}_{b_{i}}\sum_{j=1}^{n_{i}}\frac{{\left(z_{ij}^{\prime }\Gamma \right)}^{\prime }\left(y_{ij}-x_{ij}^{\prime }\beta -\xi_{1}v_{ij}\right)}{2\sigma^{-1}v_{ij}}. \end{array}$$

(3) Conditional distribution of $v_{ij}^{-1}$:

The full conditional distribution of $v_{ij}^{-1}$ follows a Inverse Gaussian(*μ*′, λ′) with p.d.f.

$$\begin{array}{c} f\left(x|\mu^{\prime },\lambda^{\prime }\right)=\sqrt{\frac{\lambda^{\prime }}{2\pi x^{3}}}\text{exp}\{\frac{-\lambda^{\prime }{\left(x-\mu^{\prime }\right)}^{2}}{2{\left(\mu^{\prime }\right)}^{2}x}\}. \end{array}$$ (10)

Here $\mu^{\prime }=\sqrt{\frac{\xi_{1}^{2}+2\xi_{2}^{2}}{\varepsilon_{ij}^{2}}},$ and $\lambda^{\prime }=\frac{\xi_{1}^{2}+2\xi_{2}^{2}}{\xi_{2}^{2}}$, where $\varepsilon_{ij}=y_{ij}-x_{ij}^{\prime }\beta -z_{ij}^{\prime }\Gamma b_{i}$.

(4) Conditional distributions of $\lambda_{1s}^{2}$ and $\lambda_{2k}^{2}$:

The full conditional distributions of $\lambda_{1s}^{2}$ and $\lambda_{2k}^{2}$ are independent Gamma distributions,

$$\begin{array}{ccc} \lambda_{1s}^{2}|t_{s},a_{\lambda_{1}},b_{\lambda_{1}} & ∼ & Gamma(1+a_{\lambda_{1}},\frac{t_{s}}{2}+b_{\lambda_{1}}),\\ \lambda_{2k}^{2}|\eta_{k},a_{\lambda_{2}},b_{\lambda_{2}} & ∼ & Gamma(\frac{k+1}{2}+a_{\lambda_{2}},\frac{\eta_{k}}{2}+b_{\lambda_{2}}). \end{array}$$

(5) Conditional distributions of *t*_*s*_ and *η*_*k*_:

The full conditional distributions of *t*_*s*_ and *η*_*k*_ are independent Inverse Gaussian,

$$\begin{array}{ccc} t_{s}^{-1}|\lambda_{1s},\beta_{s} & ∼ & Inverse\;Gaussian(\sqrt{\frac{\lambda_{1s}^{2}}{\beta_{s}^{2}}},\lambda_{1s}^{2}),\\ \eta_{k}^{-1}|\lambda_{2k},\gamma_{k} & ∼ & Inverse\;Gaussian(\sqrt{\frac{\lambda_{2k}^{2}}{\gamma_{k}^{T}\gamma_{k}}},\lambda_{2k}^{2}). \end{array}$$

(6) Conditional distribution of ***γ***:

Denote $F_{ij}=\left(b_{i}^{T}⊗z_{ij}^{\prime }\right)J_{q}$, *i* = 1, …, *n*; *j* = 1, …, *n*_*i*_, and let ***F**_ijk_* be the covariate vector corresponding to ***γ**_k_*, *k* = 1, …, *q*. The full conditional distribution of ***γ**_k_* is then a truncated normal distribution $N_{k}\left({\hat{\mu }}_{\gamma_{k}},{\hat{\Sigma }}_{\gamma_{k}}\right)I\left(\gamma_{kk}\geq 0\right)$ where

$$\begin{array}{ccc} {\hat{\Sigma }}_{\gamma_{k}} & = & {\left[\sum_{i=1}^{n}\sum_{j=1}^{n_{i}}\frac{F_{ijk}^{\prime }F_{ijk}}{2\sigma^{-1}v_{ij}}+\eta_{k}I_{k}\right]}^{-1},\\ {\hat{\mu }}_{\gamma_{k}} & = & {\hat{\Sigma }}_{\gamma_{k}}\sum_{i=1}^{n}\sum_{j=1}^{n_{i}}\frac{F_{ijk}^{\prime }\left(y_{ij}-x_{ij}^{\prime }\beta -F_{ij(k)}^{\prime }\gamma_{\left(k\right)}-\xi_{1}v_{ij}\right)}{2\sigma^{-1}v_{ij}}, \end{array}$$

where $\gamma_{\left(k\right)}={\left(\gamma_{1}^{T},\ldots ,\gamma_{k-1}^{T},\gamma_{k+1}^{T},\ldots ,\gamma_{q}^{T}\right)}^{T}$ and ***F***_*ij*(*k*_ represent the covariate matrix corresponding to *γ*_(*k*)_.

(7) Conditional distribution of *σ*:

The full conditional distribution of *σ* is

$$\begin{array}{c} Gamma(\frac{3N}{2}+a_{\sigma },\sum_{i=1}^{n}\sum_{j=1}^{n_{i}}\frac{{\left(\varepsilon_{ij}-\xi_{1}v_{ij}\right)}^{2}}{2v_{ij}}+\sum_{i=1}^{n}\sum_{j=1}^{n_{i}}v_{ij}+b_{\sigma }). \end{array}$$

## Bayesian model selection in LQMMs

The shrinkage approach proposed in the above section has potential for variable selection in LQMMs. However, the estimators are never exact 0 due to the continuous priors on ***β*** and ***γ***. This section develops a general model selection method with spike and slab priors to identify the relative important effects. We first discuss the prior specification and propose the hierarchal model. Subsequently, we consider a Partially collapsed Gibbs sampling algorithm for posterior computation, and derive the relevant conditional distributions.

We assume the following hierarchical Bayesian lasso with independent spike and slab type priors for ***β***:

$$\begin{array}{ccc} \beta_{s}|t_{s},\pi_{0s}^{\prime } & ∼ & \pi_{0s}^{\prime }\delta_{0}\left(\beta_{s}\right)+\left(1-\pi_{0s}^{\prime }\right)N\left(0,t_{s}\right),\\ t_{s}|\lambda_{1s}^{2} & ∼ & Gamma(1,\frac{\lambda_{1s}^{2}}{2}),\\ \lambda_{1s}^{2} & ∼ & Gamma(a_{\lambda_{1}},b_{\lambda_{1}}), \end{array}$$

where *δ*_0_(⋅) denotes a point mass at 0, $\pi_{0s}^{\prime }$ is the prior probability of excluding the *s*th fixed effect in the model, which is assigned a beta prior with parameters $a_{\pi_{0}^{\prime }}=1$ and $b_{\pi_{0}^{\prime }}=1$, resulting in a noninformative uniform prior on (0, 1). Furthermore, we assume the following extended hierarchical adaptive Bayesian group lasso with independent spike and slab type priors for ***γ***:

$$\begin{array}{ccc} \gamma_{k}|\eta_{k},\pi_{0} & ∼ & \pi_{0}\delta_{0}\left(\gamma_{k}\right)+\left(1-\pi_{0}\right)N_{k}\left(0,\eta_{k}I_{k}\right)I\left(\gamma_{kk}>0\right),\\ \eta_{k}|\lambda_{2k}^{2} & ∼ & Gamma(\frac{k+1}{2},\frac{\lambda_{2k}^{2}}{2}),\\ \lambda_{2k}^{2} & ∼ & Gamma(a_{\lambda_{2}},b_{\lambda_{2}}), \end{array}$$

where *π*_0_ is the prior probability of excluding the *k*th random effect in the model, which is also assumed to be a noninformative uniform prior on (0, 1). We also present the conditional distribution of unknown parameters.

(1) Conditional distribution of ***β***:

Let ${\hat{\sigma }}_{\beta_{s}}^{2}={\left[\sum_{i=1}^{n}\sum_{j=1}^{n_{i}}x_{ij}w_{ij}x_{ij}^{\prime }+t_{s}^{-1}\right]}^{-1}$, ${\hat{\mu }}_{\beta_{s}}={\hat{\sigma }}_{\beta_{s}}^{2}\sum_{i=1}^{n}\sum_{j=1}^{n_{j}}x_{ij}w_{ij}\left(y_{ij}-\xi_{1}v_{ij}\right)$ and $w_{ij}=\left(z_{ij}^{\prime }\Gamma \right){\left(z_{ij}^{\prime }\Gamma \right)}^{\prime }+\xi_{2}^{2}\sigma^{-1}v_{ij}$. The conditional distribution of *β*_*s*_ is then a spike and slab distribution,

$$\begin{array}{c} l_{0s}^{\prime }\delta_{0}\left(\beta_{s}\right)+\left(1-l_{0s}^{\prime }\right)N\left({\hat{\mu }}_{\beta_{s}},{\hat{\sigma }}_{\beta_{s}}^{2}\right), \end{array}$$

where

$$\begin{array}{c} l_{0s}^{\prime }=\frac{\pi_{0s}^{\prime }}{\pi_{0s}^{\prime }+\left(1-\pi_{0s}^{\prime }\right)\sqrt{\frac{2{\hat{\sigma }}_{\beta_{s}}^{2}}{t_{s}}}exp\{\frac{{\hat{\mu }}_{\beta_{s}}^{2}}{2{\hat{\sigma }}_{\beta_{s}}^{2}}\}}. \end{array}$$

(2) Conditional distribution of ***b**_i_*:

The conditional distribution of ***b**_i_* is a multivariate normal distribution with mean ${\hat{\mu }}_{b_{i}}$ and variance ${\hat{\Sigma }}_{b_{i}}$ where

$$\begin{array}{c} {\hat{\Sigma }}_{b_{i}}={\left[\sum_{j=1}^{n_{i}}\frac{{\left(z_{ij}^{\prime }\Gamma \right)}^{\prime }\left(z_{ij}^{\prime }\Gamma \right)}{2\sigma^{-1}v_{ij}}+I_{q}\right]}^{-1},\;\;{\hat{\mu }}_{b_{i}}={\hat{\Sigma }}_{b_{i}}\sum_{j=1}^{n_{i}}\frac{{\left(z_{ij}^{\prime }\Gamma \right)}^{\prime }\left(y_{ij}-x_{ij}^{\prime }\beta -\xi_{1}v_{ij}\right)}{2\sigma^{-1}v_{ij}}. \end{array}$$

(3) Conditional distribution of $v_{ij}^{-1}$:

The full conditional distribution of $v_{ij}^{-1}$ follows a Inverse Gaussian(*μ*′, λ′) with $\mu^{\prime }=\sqrt{\frac{\xi_{1}^{2}+2\xi_{2}^{2}}{\varepsilon_{ij}^{2}}}$ and $\lambda^{\prime }=\frac{\xi_{1}^{2}+2\xi_{2}^{2}}{\xi_{2}^{2}}$, where $\varepsilon_{ij}=y_{ij}-x_{ij}^{\prime }\beta -z_{ij}^{\prime }\Gamma b_{i}$.

(4) Conditional distributions of λ_1*s*_ and λ_2*k*_:

The full conditional distributions of λ_1*s*_ and λ_2*k*_ are independent Gamma distributions,

$$\begin{array}{ccc} \lambda_{1s}^{2}|t_{s},a_{\lambda_{1}},b_{\lambda_{1}} & ∼ & Gamma(1+a_{\lambda_{1}},\frac{t_{s}}{2}+b_{\lambda_{1}}),\\ \lambda_{2k}^{2}|\eta_{k},a_{\lambda_{2}},b_{\lambda_{2}} & ∼ & Gamma(\frac{k+1}{2}+a_{\lambda_{2}},\frac{\eta_{k}}{2}+b_{\lambda_{2}}). \end{array}$$

(5) Conditional distributions of *t*_*s*_ and *η*_*k*_:

$$\begin{array}{c} t_{s}^{-1}|\lambda_{1s},\beta_{s}∼\{\begin{array}{c} Inverse\;Gamma\left(1,\frac{\lambda_{1s}^{2}}{2}\right),\text{if}\;\beta_{s}=0,\\ Inverse\;Gaussian\left(\sqrt{\frac{\lambda_{1s}^{2}}{\beta_{s}^{2}}},\lambda_{1s}^{2}\right),\text{if}\;\beta_{s}\neq 0, \end{array} \end{array}$$

$$\begin{array}{c} \eta_{k}^{-1}|\lambda_{2k},\gamma_{k}∼\{\begin{array}{c} Inverse\;Gamma\left(1,\frac{\lambda_{2k}^{2}}{2}\right),\text{if}\;\gamma_{k}=0,\\ Inverse\;Gaussian\left(\sqrt{\frac{\lambda_{2k}^{2}}{\gamma_{k}^{T}\gamma_{k}}},\lambda_{2k}^{2}\right),\text{if}\;\gamma_{k}\neq 0, \end{array} \end{array}$$

where *InverseGamma*(*a*, *b*) denotes a Inverse Gamma distribution with shape parameter *a* and scale parameter *b*.

(6) Conditional distribution of $\pi_{0s}^{\prime }$ and *π*_0_:

The full conditional distributions of $\pi_{0s}^{\prime }$ and *π*_0_ are independent Beta distribution.

$$\begin{array}{ccc} \pi_{0s}^{\prime }|\beta_{s} & ∼ & beta(1+I\left(\beta_{s}=0\right),2-I\left(\beta_{s}=0\right)),\\ \pi_{0}|\gamma & ∼ & beta(1+\sum_{k=1}^{q}I\left(\gamma_{k}=0\right),1+q-\sum_{k=1}^{q}I\left(\gamma_{k}=0\right)). \end{array}$$

(7) Conditional distribution of ***γ***:

Denote $F_{ij}=\left(b_{i}^{T}⊗z_{ij}^{\prime }\right)J_{q}$, ${\hat{\Sigma }}_{\gamma_{k}}={\left[\sum_{i=1}^{n}\sum_{j=1}^{n_{i}}\frac{F_{ijk}^{\prime }F_{ijk}}{2\sigma^{-1}v_{ij}}+\eta_{k}I_{k}\right]}^{-1}$, ${\hat{\mu }}_{\gamma_{k}}={\hat{\Sigma }}_{\gamma_{k}}\sum_{i=1}^{n}\sum_{j=1}^{n_{i}}\frac{F_{ijk}^{\prime }\left(y_{ij}-x_{ij}^{\prime }\beta -F_{ij(k)}^{\prime }\gamma_{\left(k\right)}-\xi_{1}v_{ij}\right)}{2\sigma^{-1}v_{ij}}$, and let ***F**_ijk_* be the covariate vector corresponding to be the covariate vector corresponding to ***γ**_k_*, *k* = 1, …, *q*. The full conditional distribution of ***γ**_k_* is then a a spike and slab distribution,

$$\begin{array}{c} l_{0k}\delta_{0}\left(\gamma_{k}\right)+\left(1-l_{0k}\right)N_{k}\left({\hat{\mu }}_{\gamma_{k}},{\hat{\Sigma }}_{\gamma_{k}}\right)I\left(\gamma_{kk}\geq 0\right), \end{array}$$

where

$$\begin{array}{c} l_{0k}=\frac{\pi_{0k}}{\pi_{0k}+2\left(1-\pi_{0k}\right)\eta_{k}^{-\frac{k}{2}}{\left|{\hat{\Sigma }}_{\gamma_{k}}\right|}^{\frac{1}{2}}\Phi \left(\frac{{\hat{\mu }}_{kk}}{{\hat{\sigma }}_{kk}}\right)exp\{\frac{{\hat{\mu }}_{\gamma_{k}}^{\prime }{\hat{\Sigma }}_{\gamma_{k}}^{-1}{\hat{\mu }}_{\gamma_{k}}}{2}\}}. \end{array}$$

Here, **Φ**(⋅) indicates the cumulative distribution function of the standard normal distribution, ${\hat{\mu }}_{kk}$ is the *k*th element of ${\hat{\mu }}_{\gamma_{k}}$ and ${\hat{\sigma }}_{kk}^{2}$ is the *k*th diagonal element of ${\hat{\Sigma }}_{\gamma_{k}}$.

(8) Conditional distribution of *σ*:

The full conditional distribution of *σ* is

$$Gamma(\frac{3N}{2}+a_{\sigma },\sum_{i=1}^{n}\sum_{j=1}^{n_{i}}\frac{{\left(\varepsilon_{ij}-\xi_{1}v_{ij}\right)}^{2}}{2v_{ij}}+\sum_{i=1}^{n}\sum_{j=1}^{n_{i}}v_{ij}+b_{\sigma }).$$

## Simulation studies

In this section we check the performance of the proposed extended Bayesian adaptive group lasso approach (adGL) and the Bayesian adaptive group lasso with independent spike and slab type priors approach (adSpikeGL) through simulations. We also compare them with other models: the PMQ method reported by [10], the BL and BAL methods proposed by [11] and the ordinary quantile regression estimator (QR) focusing solely on independent regression. The test data are generated from the following model:

$$\begin{array}{c} y_{ij}=x_{ij}^{\prime }\beta +z_{ij}^{\prime }\alpha_{i}+\varepsilon_{ij}, \end{array}$$

for *i* = 1, …, 50, *j* = 1, …, 5, where *ε*_*ij*_ has the *τ*th quantile equal to 0, random effects ***α**_i_* ∼ *N*(0, ***D***), where

$$\begin{array}{c} D={\left(\begin{array}{cc} D_{1} & 0\\ 0 & 0 \end{array}\right)}_{8\times 8},D_{1}=(\begin{array}{ccc} 9 & 4.8 & 0.6\\ 4.8 & 4 & 1\\ 0.6 & 1 & 1 \end{array}) \end{array}$$ (11)

Similar formulations of ***D*** in mixed models have been used by [29] and [30]. We set $x_{ij}^{\prime }=\left(x_{ij1},\ldots ,x_{ij8}\right)$ and $z_{ij}^{\prime }=x_{ij}^{\prime }$, where *x*_*ijk*_, *k* = 1, …, 8 are drawn independently from the uniform [−2, 2]. The fixed effects parameters ***β*** = (*β*_1_, …, *β*_8_) are set as follows:

- **Simulation 1**: ***β*** = (3, 1.5, 0, 0, 2, 0, 0, 0)′ to illustrate a sparse case;
- **Simulation 2**: ***β*** = (0.85, 0.85, 0.85, 0.85, 0.85, 0.85, 0.85, 0.85)′ to illustrate a dense case;
- **Simulation 3**: ***β*** = (5, 0, 0, 0, 0, 0, 0, 0)′ to illustrate a very sparse case.

With each simulation of ***β***, we generate data under three different quantiles i.e. *τ* = 0.1, 0.3 and 0.5, and four different error distributions: normal, normal mixture, laplace, and laplace mixture as follows

**Fig 1:**
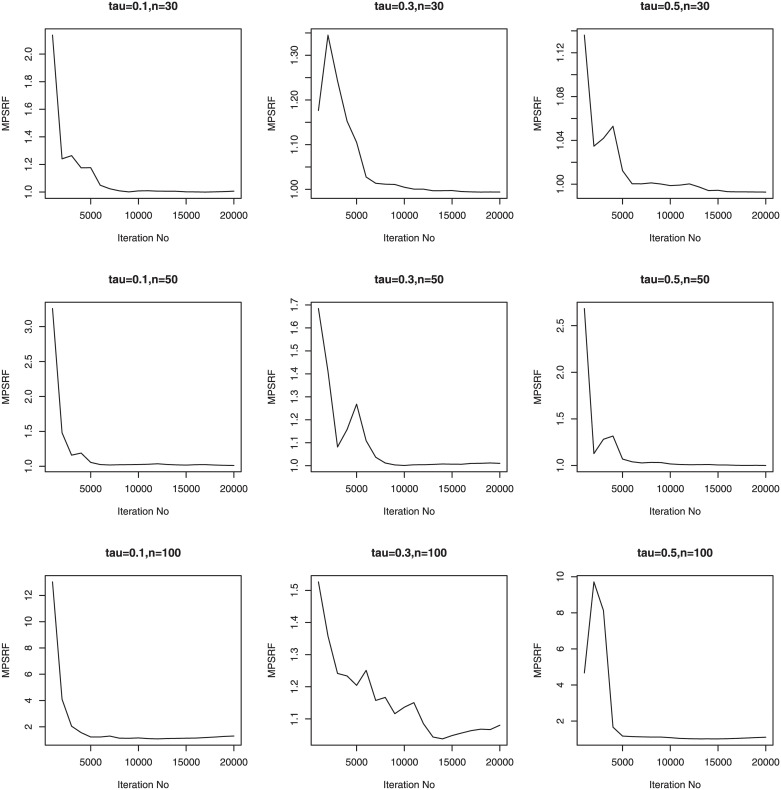
MPSRF for adGL in Simulation 1, when normal mixture error distribution.

**Fig 2:**
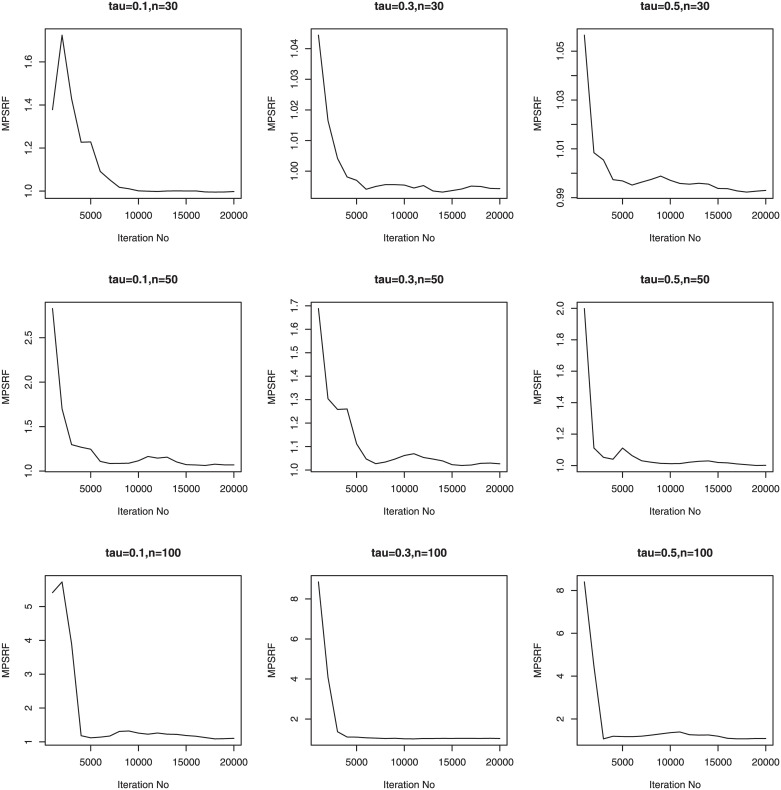
MPSRF for adGL in Simulation 2, when normal mixture error distribution.

**Fig 3:**
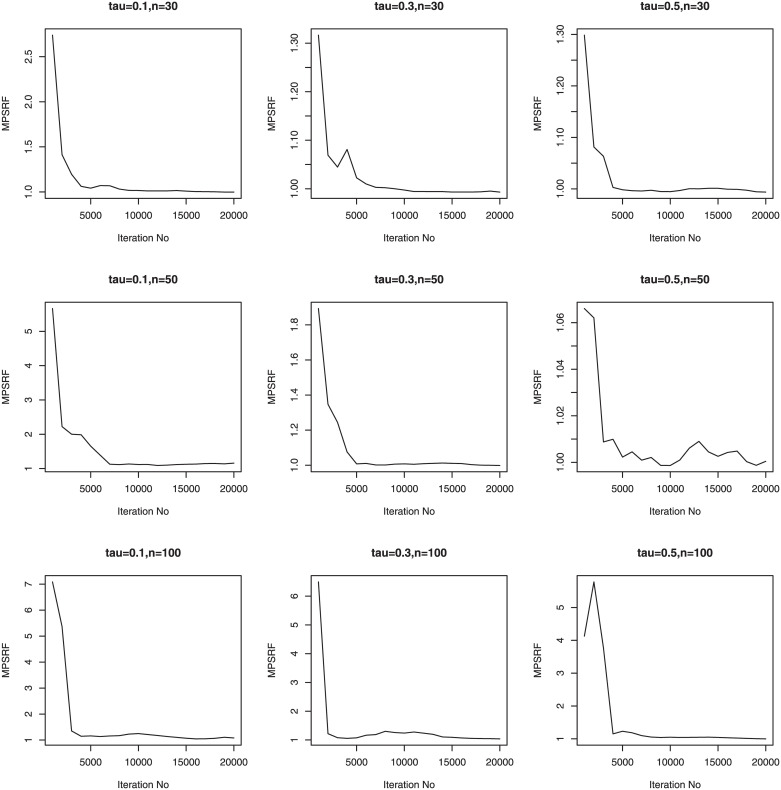
MPSRF for adGL in Simulation 3, when normal mixture error distribution.

**Fig 4:**
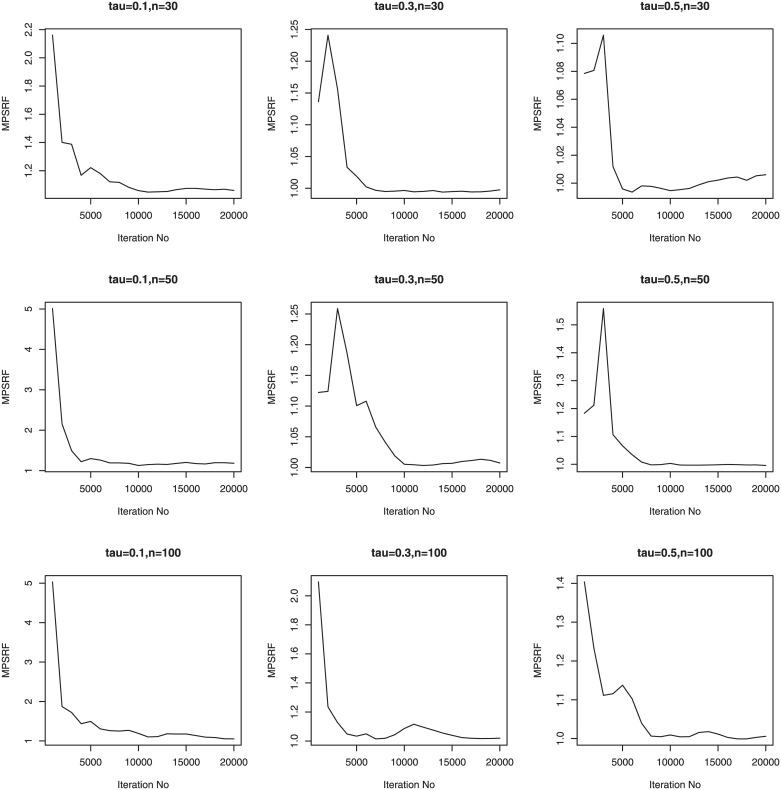
MPSRF for adSpikeGL in Simulation 1, when normal mixture error distribution.

**Fig 5:**
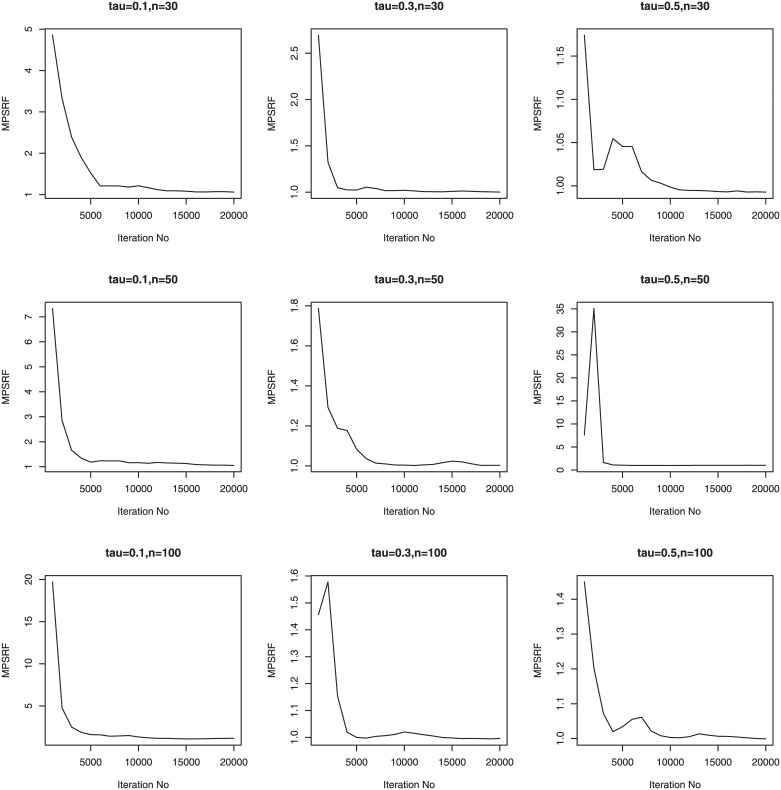
MPSRF for adSpikeGL in Simulation 2, when normal mixture error distribution.

**Fig 6:**
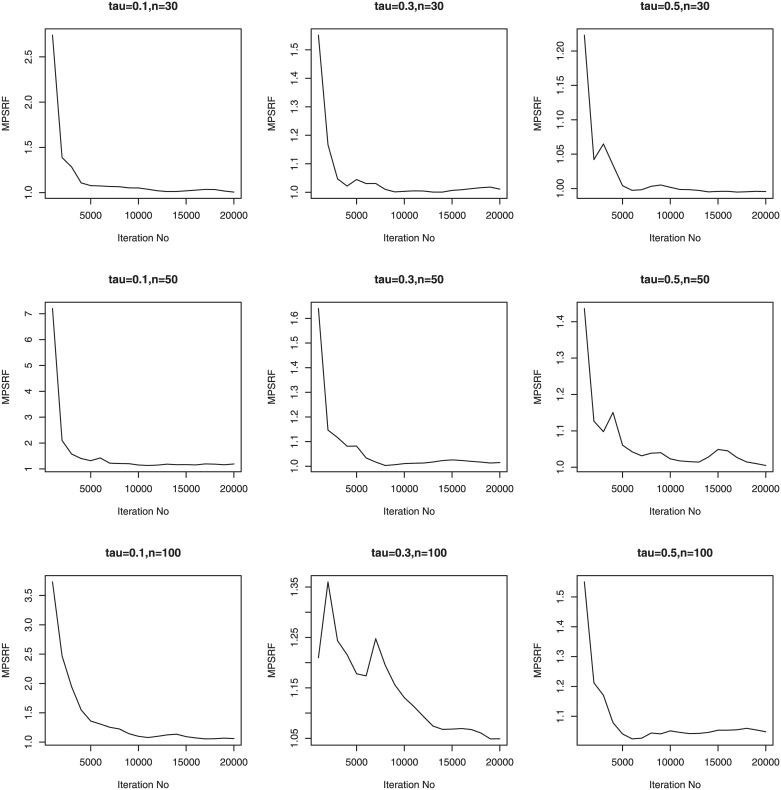
MPSRF for adSpikeGL in Simulation 3, when normal mixture error distribution.

- (1) *ε*_*ij*_ ∼ *N*(0, 9), *i* = 1, …, *n*, *j* = 1, …, *n*_*i*_;
- (2) *ε*_*ij*_ ∼ 0.1*N*(0, 1) + 0.9*N*(0, 5), *i* = 1, …, *n*, *j* = 1, …, *n*_*i*_;
- (3) *ε*_*ij*_ ∼ *Laplace*(0, 3), *i* = 1, …, *n*, *j* = 1, …, *n*_*i*_;
- (4) $\varepsilon_{ij}∼0.1Laplace\left(0,1\right)+0.9Laplace\left(0,\sqrt{5}\right),i=1,\ldots ,n,j=1,\ldots ,n_{i}$;

We consider three sample sizes *n* = 30, *n*_*i*_ = 5; *n* = 50, *n*_*i*_ = 5; *n* = 100, *n*_*i*_ = 5. Therefore, we have a total of 36 simulation setups for each of the sample sizes. We simulate 100 data sets for simulation setups. Priors for the Bayesian methods are taken to be weak prior. The hyperparameters in the Gamma priors for tuning and scale parameters are all set to be 0.1. We run our PCG sampler for 20000 following initial burn-in 10000 cycles. The convergence of the proposed PCG is monitored by using the multivariate potential scale reduction factor (MPSRF) introduced by [31]. Figs 1–6 show that the MPSRF generally become stable and get close to 1 after about 10000 iterations under normal mixture error distribution, suggesting that the above burn-in size is large enough to ensure the convergence of PCG. The results for other error distributions are similar and omitted.

**Fig 7:**
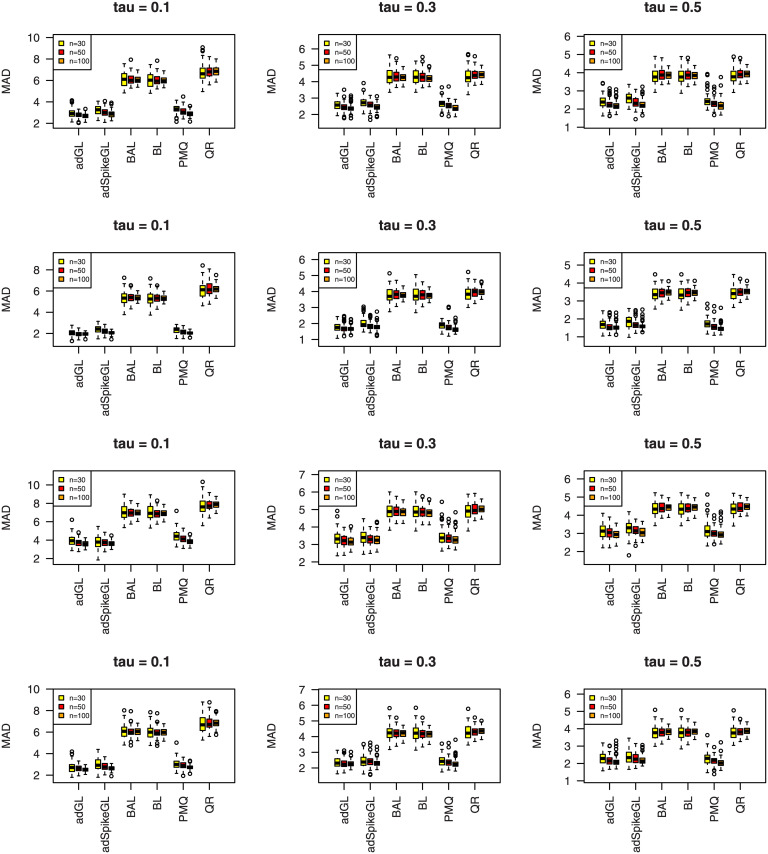
Boxplots summarizing the MAD for three sample sizes in Simulation 1. The rows from top to bottom correspond to normal, normal mixture, laplace, and laplace mixture error distributions respectively.

**Fig 8:**
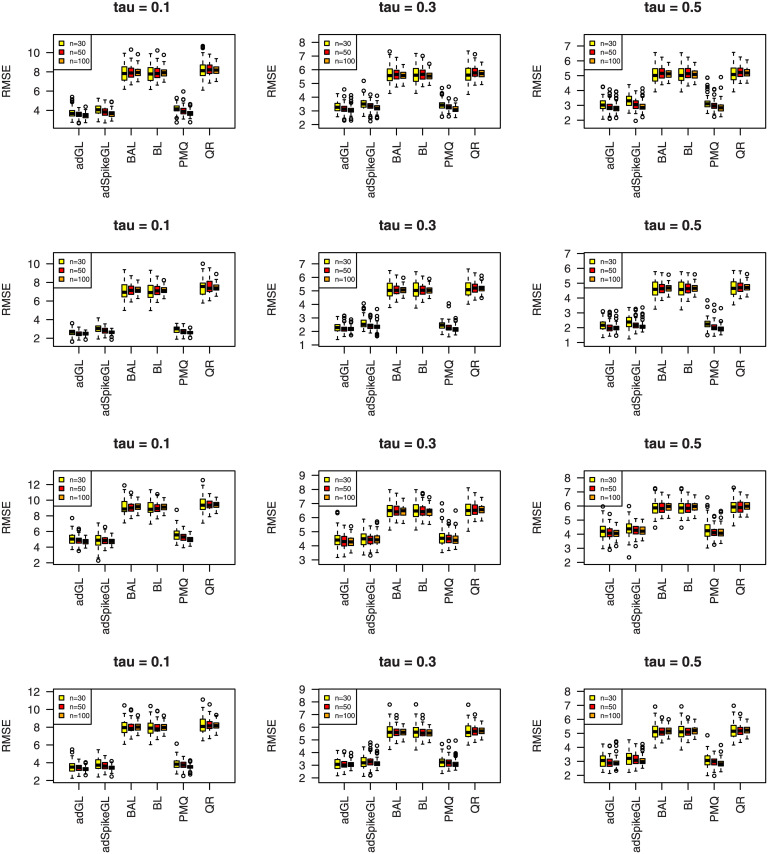
Boxplots summarizing the RMSE for three sample sizes in Simulation 1. The rows from top to bottom correspond to normal, normal mixture, laplace, and laplace mixture error distributions respectively.

**Fig 9:**
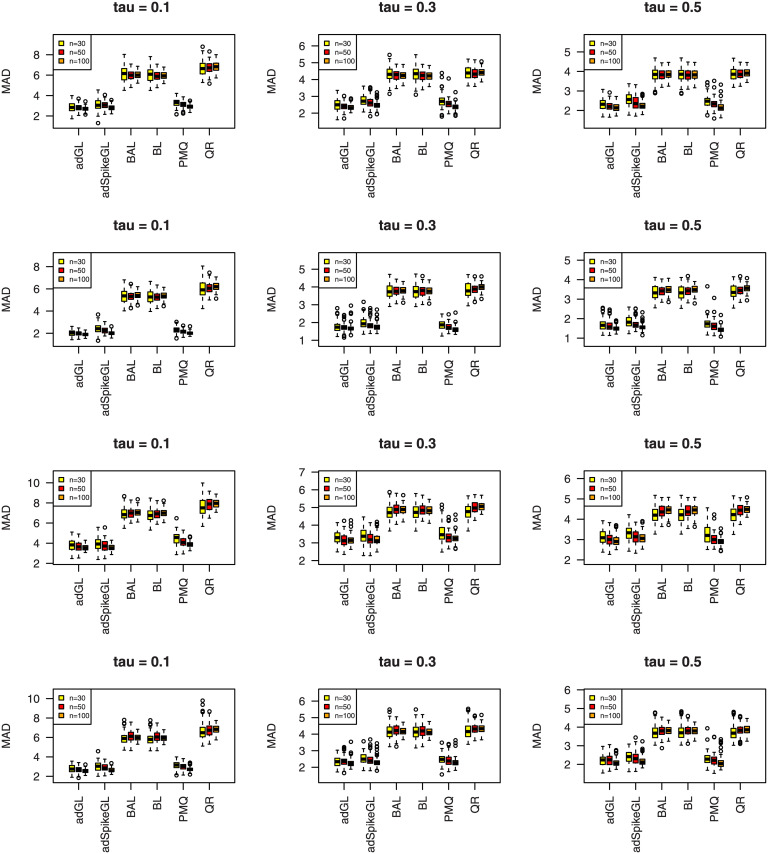
Boxplots summarizing the MAD for three sample sizes in Simulation 2. The rows from top to bottom correspond to normal, normal mixture, laplace, and laplace mixture error distributions respectively.

**Fig 10:**
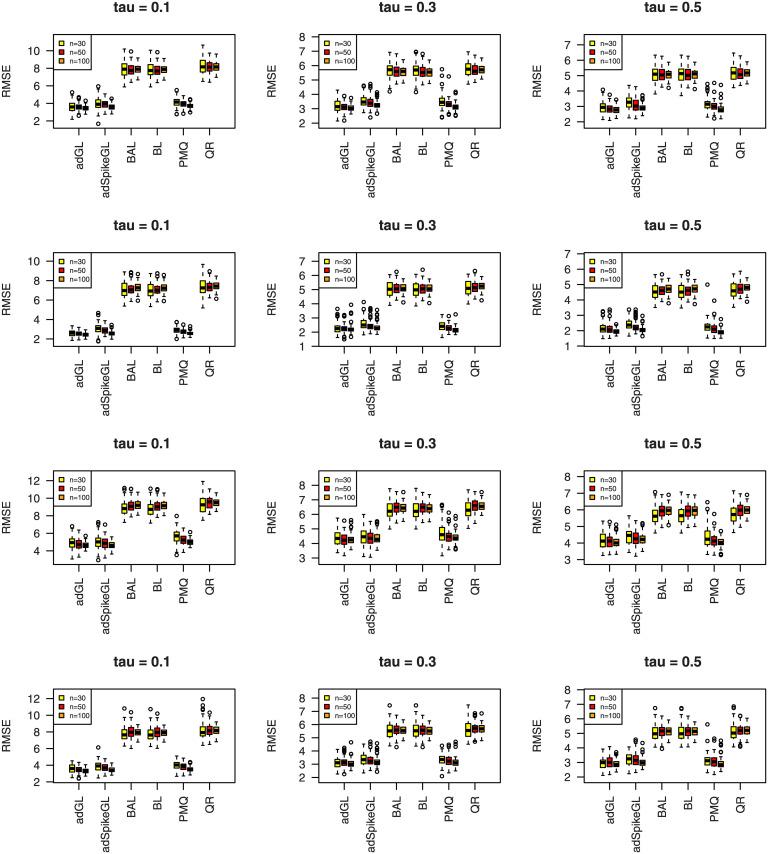
Boxplots summarizing the RMSE for three sample sizes in Simulation 2. The rows from top to bottom correspond to normal, normal mixture, laplace, and laplace mixture error distributions respectively.

**Fig 11:**
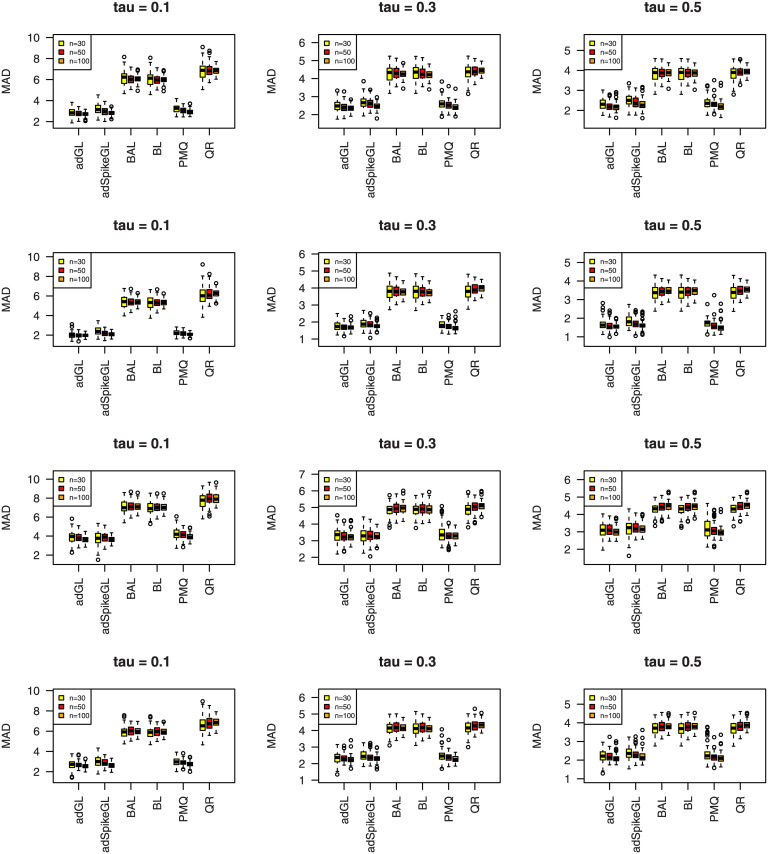
Boxplots summarizing the MAD for three sample sizes in Simulation 3. The rows from top to bottom correspond to normal, normal mixture, laplace, and laplace mixture error distributions respectively.

**Fig 12:**
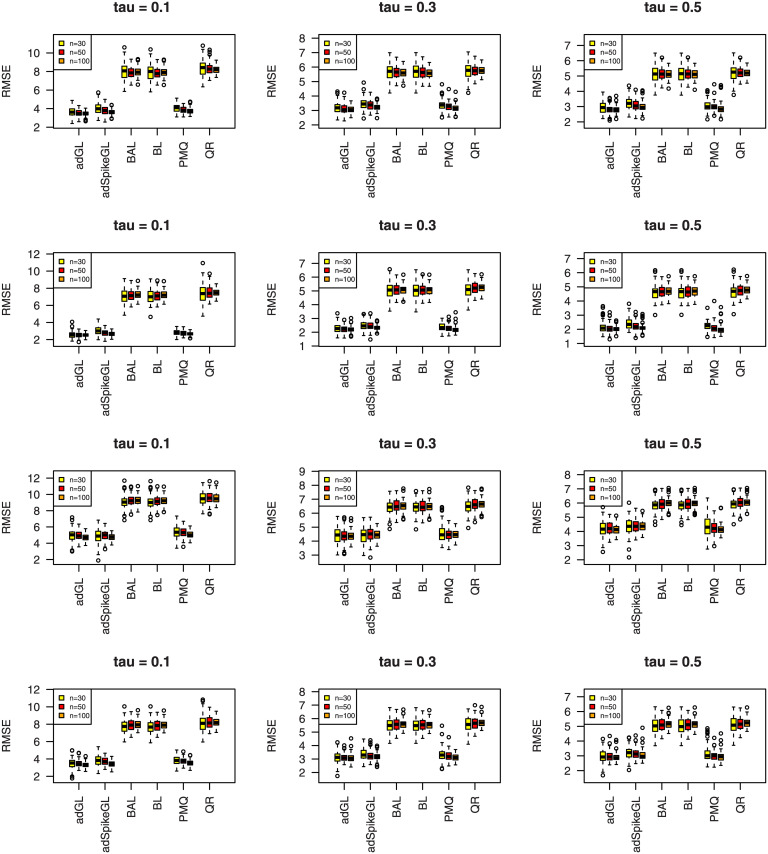
Boxplots summarizing the RMSE for three sample sizes in Simulation 3. The rows from top to bottom correspond to normal, normal mixture, laplace, and laplace mixture error distributions respectively.

We use the posterior median estimates of ***β*** and ***D*** as our point estimator, denoted as $\hat{\beta }$ and $\hat{D}$, respectively, and we consider two error measures including the mean absolute deviations (MAD), root mean squared errors (RMSE). To check the performance of the parameter estimation, we also calculate the median of mean square error for ***β*** ($MME(\hat{\beta })$) and the median of quadratic loss error for ***D*** ($MMR(\hat{D})$). More specifically,

$$\begin{array}{ccc} MAD & = & \frac{1}{250}\sum_{i=1}^{50}\sum_{j=1}^{5}\left|y_{ij}-{\hat{y}}_{ij}\right|,\\ RMSE & = & \sqrt{\frac{1}{250}\sum_{i=1}^{50}\sum_{j=1}^{5}{\left(y_{ij}-{\hat{y}}_{ij}\right)}^{2}},\\ ME(\hat{\beta }) & = & {\left(\hat{\beta }-\beta \right)}^{T}\left(\hat{\beta }-\beta \right),\\ MR(\hat{D}) & = & \text{trace}{\left[{\left(\hat{D}-D\right)}^{2}\right]}^{1/2}, \end{array}$$

where ${\hat{y}}_{ij}$ is the predicted value of *y*_*ij*_, and the median is taken over the 100 simulations. The results of MAD and RMSE are summarized in Figs 7–12, meanwhile $MME(\hat{\beta })$ and $MMR(\hat{D})$ are listed in Tables 1–9. Because BL, BAL, and QR neglect the covariance structure of random effects, we just report the $MMR(\hat{D})$ results of the other three techniques for comparison.

**Table 1:** Median of mean square errors ($MME(\hat{\beta })$) and quadratic loss errors($MMR(\hat{D})$) at *τ* = 0.1 for Simulation 1. $MMR(\hat{D})$ are in parentheses.

Sample size	Method	Error distribution			
		*Normal*	*Normalmixtrue*	*Laplace*	*Laplacemixtrue*
n = 30	adGL	**3.17**(11.314)	1.783(9.209)	**4.034**(14.133)	**2.273**(11.656)
	adSpikeGL	3.535(**11.089**)	**1.475**(**9.02**)	6.26(20.161)	2.409(12.694)
	PMQ	5.017(11.124)	4.127(9.899)	5.494(**14.108**)	4.364(12.485)
	BL	5.635(-)	5.919(-)	6.404(-)	7.011(-)
	BAL	5.377(-)	5.708(-)	5.61(-)	6.155(-)
	QR	9.964(-)	11.953(-)	10.803(-)	12.641(-)
n = 50	adGL	2.645(**9.065**)	1.508(7.383)	3.508(12.752)	2.002(9.291)
	adSpikeGL	**2.102**(9.102)	**0.944**(**6.497**)	3.443(14.761)	1.627(9.121)
	PMQ	2.643(8.517)	1.796(6.588)	**3.151**(**11.198**)	2.089(**8.981**)
	BL	4.422(-)	5.452(-)	4.114(-)	5.35(-)
	BAL	4.011(-)	4.763(-)	3.661(-)	4.736(-)
	QR	7.875(-)	10.489(-)	8.198(-)	10.686(-)
n = 100	adGL	2.125(7.452)	1.133(5.182)	3.094(11.117)	1.481(7.841)
	adSpikeGL	**1.601**(6.809)	**0.802**(**4.374**)	2.692(12.212)	1.164(7.423)
	PMQ	1.825(**6.597**)	1.019(4.745)	**2.526**(**10.248**)	1.192(**6.914**)
	BL	3.627(-)	4.737(-)	3.462(-)	4.885(-)
	BAL	3.2(-)	4.125(-)	2.923(-)	4.21(-)
	QR	7.128(-)	9.408(-)	7.445(-)	9.278(-)

**Table 2:** Median of mean square errors($MME(\hat{\beta })$) and quadratic loss errors($MMR(\hat{D})$) at *τ* = 0.3 for Simulation 1. $MMR(\hat{D})$ are in parentheses.

Sample size	Method	Error distribution			
		*Normal*	*Normalmixtrue*	*Laplace*	*Laplacemixtrue*
n = 30	adGL	**0.958**(11.408)	**0.607**(9.013)	**1.447**(13.115)	**0.949**(10.876)
	adSpikeGL	1.394(**7.827**)	0.85(**7.501**)	2.456(**11.005**)	1.572(**9.061**)
	PMQ	4.366(10.095)	3.825(10.022)	5.127(13.1)	4.299(11.501)
	BL	2.838(-)	2.763(-)	3.785(-)	3.697(-)
	BAL	2.576(-)	2.64(-)	3.761(-)	3.615(-)
	QR	3.001(-)	3.068(-)	3.866(-)	3.842(-)
n = 50	adGL	0.543(8.77)	0.357(7.495)	0.736(10.75)	0.516(8.6)
	adSpikeGL	**0.337**(**5.917**)	**0.241**(**4.7**)	**0.521**(**7.668**)	**0.432**(**6.034**)
	PMQ	1.395(6.996)	0.975(5.921)	1.402(8.938)	1.441(6.762)
	BL	1.743(-)	1.974(-)	2.748(-)	2.54(-)
	BAL	1.736(-)	1.863(-)	2.637(-)	2.506(-)
	QR	1.978(-)	2.062(-)	2.862(-)	2.606(-)
n = 100	adGL	0.3(6.429)	0.191(5.149)	0.441(8.083)	0.313(6.55)
	adSpikeGL	**0.198**(**4.973**)	**0.133**(**3.585**)	**0.298**(**6.177**)	**0.28**(**3.93**)
	PMQ	0.373(5.299)	0.305(3.939)	0.576(6.255)	0.388(4.797)
	BL	1.289(-)	1.567(-)	1.951(-)	2.109(-)
	BAL	1.243(-)	1.469(-)	1.859(-)	1.984(-)
	QR	1.368(-)	1.544(-)	2.095(-)	2.1(-)

**Table 3:** Median of mean square errors($MME(\hat{\beta })$) and quadratic loss errors($MMR(\hat{D})$) at *τ* = 0.5 for Simulation 1. $MMR(\hat{D})$ are in parentheses.

Sample size	Method	Error distribution			
		*Normal*	*Normalmixtrue*	*Laplace*	*Laplacemixtrue*
n = 30	adGL	**0.924**(11.166)	**0.581**(8.938)	**1.278**(13.494)	**0.862**(11.367)
	adSpikeGL	1.377(**7.44**)	0.732(**6.637**)	1.736(**8.771**)	1.181(**7.687**)
	PMQ	4.216(10.914)	3.594(10.373)	4.813(13.136)	4.121(10.363)
	BL	1.729(-)	1.276(-)	1.968(-)	1.596(-)
	BAL	1.734(-)	1.289(-)	1.923(-)	1.633(-)
	QR	2.032(-)	1.517(-)	2.223(-)	1.88(-)
n = 50	adGL	0.421(8.836)	0.326(7.586)	0.605(10.555)	0.433(8.434)
	adSpikeGL	**0.317**(**6.044**)	**0.238**(**5.088**)	**0.399**(**6.996**)	**0.283**(**6.042**)
	PMQ	1.327(7.136)	0.993(5.648)	1.324(7.431)	1.409(6.689)
	BL	0.982(-)	0.823(-)	1.086(-)	0.912(-)
	BAL	1.007(-)	0.831(-)	1.109(-)	0.906(-)
	QR	1.157(-)	0.923(-)	1.294(-)	1.102(-)
n = 100	adGL	0.286(6.651)	0.18(5.065)	0.31(7.512)	0.207(6.058)
	adSpikeGL	**0.197**(**4.55**)	**0.128**(**3.223**)	**0.22**(**5.378**)	**0.134**(**4.589**)
	PMQ	0.344(5.167)	0.296(3.711)	0.472(5.622)	0.3(4.821)
	BL	0.471(-)	0.402(-)	0.573(-)	0.469(-)
	BAL	0.5(-)	0.423(-)	0.582(-)	0.478(-)
	QR	0.541(-)	0.456(-)	0.634(-)	0.515(-)

**Table 4:** Median of mean square errors($MME(\hat{\beta })$) and quadratic loss errors($MMR(\hat{D})$) at *τ* = 0.1 for Simulation 2. $MMR(\hat{D})$ are in parentheses.

Sample size	Method	Error distribution			
		*Normal*	*Normalmixtrue*	*Laplace*	*Laplacemixtrue*
n = 30	adGL	3.28(12.016)	2.091(9.127)	4.671(14.802)	2.492(10.868)
	adSpikeGL	4.802(13.526)	2.63(8.487)	7.702(18.516)	3.663(12.025)
	PMQ	**2.796**(**9.328**)	**1.919**(**7.995**)	**3.461**(**11.497**)	**2.477**(**8.897**)
	BL	5.359(-)	6.499(-)	5.447(-)	6.328(-)
	BAL	5.214(-)	5.98(-)	5.255(-)	5.842(-)
	QR	9.121(-)	11.535(-)	10.766(-)	11.09(-)
n = 50	adGL	2.639(9.979)	1.541(7.654)	3.914(13.945)	2.098(10.088)
	adSpikeGL	3.117(9.197)	1.909(6.214)	4.98(16.776)	2.641(10.728)
	PMQ	**2.19**(**8.009**)	**1.426**(**6.192**)	**2.884**(**12.507**)	**1.769**(**8.396**)
	BL	4.36(-)	5.097(-)	4.458(-)	5.872(-)
	BAL	3.97(-)	4.407(-)	4.165(-)	5.476(-)
	QR	8.105(-)	9.584(-)	9.089(-)	10.387(-)
n = 100	adGL	2.23(7.876)	1.279(5.596)	3.163(12.262)	1.446(7.97)
	adSpikeGL	2.364(6.951)	1.296(5.048)	3.708(13.513)	1.772(8.228)
	PMQ	**1.796**(**6.807**)	**1.097**(**4.709**)	**2.515**(**10.873**)	**1.247**(**7.199**)
	BL	3.476(-)	5.193(-)	3.526(-)	4.909(-)
	BAL	3.106(-)	4.431(-)	2.995(-)	4.17(-)
	QR	7.104(-)	9.498(-)	7.716(-)	9.371(-)

**Table 5:** Median of mean square errors($MME(\hat{\beta })$) and quadratic loss errors($MMR(\hat{D})$) at *τ* = 0.3 for Simulation 2. $MMR(\hat{D})$ are in parentheses.

Sample size	Method	Error distribution			
		*Normal*	*Normalmixtrue*	*Laplace*	*Laplacemixtrue*
n = 30	adGL	**1.337**(11.777)	**0.948**(8.895)	**1.836**(13.974)	**1.212**(10.574)
	adSpikeGL	3.095(10.405)	1.849(**7.039**)	4.022(13.495)	2.494(8.874)
	PMQ	1.985(**10.15**)	1.423(7.629)	2.495(**11.006**)	1.765(**7.828**)
	BL	2.722(-)	2.548(-)	3.531(-)	3.35(-)
	BAL	2.678(-)	2.477(-)	3.514(-)	3.323(-)
	QR	3.2(-)	2.831(-)	3.648(-)	3.539(-)
n = 50	adGL	**0.773**(9.367)	**0.51**(7.751)	**1.092**(10.676)	**0.837**(8.88)
	adSpikeGL	1.78(**6.842**)	1.363(**5.378**)	2.569(8.632)	1.692(**5.98**)
	PMQ	1.091(7.026)	0.819(5.406)	1.614(**8.468**)	1.113(6.712)
	BL	1.831(-)	1.923(-)	2.918(-)	2.76(-)
	BAL	1.787(-)	1.874(-)	2.848(-)	2.702(-)
	QR	1.989(-)	1.976(-)	3.115(-)	2.905(-)
n = 100	adGL	**0.37**(6.871)	**0.225**(5.369)	**0.561**(7.846)	**0.328**(6.736)
	adSpikeGL	0.871(**4.78**)	0.546(**3.421**)	1.137(**5.862**)	0.789(**4.451**)
	PMQ	0.515(5.01)	0.347(3.739)	0.703(6.179)	0.468(5.179)
	BL	1.253(-)	1.61(-)	1.952(-)	2.022(-)
	BAL	1.183(-)	1.483(-)	1.848(-)	1.956(-)
	QR	1.339(-)	1.627(-)	2.083(-)	2.098(-)

**Table 6:** Median of mean square errors($MME(\hat{\beta })$) and quadratic loss errors($MMR(\hat{D})$) at *τ* = 0.5 for Simulation 2. $MMR(\hat{D})$ are in parentheses.

Sample size	Method	Error distribution			
		*Normal*	*Normalmixtrue*	*Laplace*	*Laplacemixtrue*
n = 30	adGL	**1.242**(11.082)	**0.903**(9.625)	**1.544**(13.627)	**1.089**(10.873)
	adSpikeGL	2.824(**8.786**)	1.797(**6.892**)	3.649(**10.828**)	2.247(8.175)
	PMQ	1.894(9.806)	1.379(7.22)	2.419(11.003)	1.6(**7.938**)
	BL	1.668(-)	1.266(-)	2.046(-)	1.502(-)
	BAL	1.648(-)	1.267(-)	2.005(-)	1.508(-)
	QR	2.074(-)	1.406(-)	2.384(-)	1.864(-)
n = 50	adGL	**0.74**(9.271)	**0.502**(7.87)	**0.953**(10.308)	**0.667**(8.587)
	adSpikeGL	1.636(6.982)	1.288(**5.22**)	2.221(**7.434**)	1.581(**6.003**)
	PMQ	1.03(**6.806**)	0.802(5.529)	1.352(7.545)	0.99(6.257)
	BL	1.023(-)	0.845(-)	1.298(-)	0.945(-)
	BAL	1.008(-)	0.871(-)	1.312(-)	0.923(-)
	QR	1.121(-)	0.959(-)	1.528(-)	1.007(-)
n = 100	adGL	**0.33**(6.845)	**0.213**(5.253)	**0.422**(7.898)	**0.265**(6.053)
	adSpikeGL	0.843(**4.707**)	0.644(**3.548**)	0.948(**5.46**)	0.77(**4.39**)
	PMQ	0.504(5.029)	0.334(3.65)	0.58(5.615)	0.412(4.861)
	BL	0.517(-)	0.363(-)	0.57(-)	0.429(-)
	BAL	0.536(-)	0.389(-)	0.554(-)	0.43(-)
	QR	0.55(-)	0.446(-)	0.632(-)	0.469(-)

**Table 7:** Median of mean square errors($MME(\hat{\beta })$) and quadratic loss errors($MMR(\hat{D})$) at *τ* = 0.1 for Simulation 3. $MMR(\hat{D})$ are in parentheses.

Sample size	Method	Error distribution			
		*Normal*	*Normalmixtrue*	*Laplace*	*Laplacemixtrue*
n = 30	adGL	2.922(11.216)	1.577(9.453)	3.801(14.215)	1.962(11.422)
	adSpikeGL	**1.284**(11.385)	**0.509**(**7.829**)	**3.764**(15.632)	**1.044**(10.661)
	PMQ	2.045(**9.833**)	1.358(8.179)	**3.375**(**12.93**)	1.861(**9.465**)
	BL	5.425(-)	6.815(-)	6.204(-)	6.326(-)
	BAL	5.426(-)	6.208(-)	6.357(-)	5.936(-)
	QR	9.733(-)	11.759(-)	11.056(-)	10.68(-)
n = 50	adGL	2.584(9.042)	1.231(7.481)	2.929(12.619)	1.639(9.513)
	adSpikeGL	**1.545**(8.405)	**0.616**(**6.177**)	2.372(14.616)	**1.079**(9.174)
	PMQ	1.931(**7.836**)	1.098(6.659)	**2.336**(**10.67**)	1.295(**8.154**)
	BL	4.411(-)	5.516(-)	4.747(-)	5.774(-)
	BAL	3.947(-)	5.067(-)	4.248(-)	5.313(-)
	QR	8.331(-)	10.489(-)	9.016(-)	11.041(-)
n = 100	adGL	2.09(7.332)	1.033(5.836)	2.619(11.603)	1.399(7.948)
	adSpikeGL	**1.538**(6.93)	**0.632**(5.038)	2.104(13.169)	**1.03**(7.819)
	PMQ	1.662(**6.521**)	0.842(**4.998**)	**2.018**(**10.587**)	1.097(**7.047**)
	BL	3.592(-)	5.101(-)	3.939(-)	4.656(-)
	BAL	3.106(-)	4.404(-)	3.438(-)	4.084(-)
	QR	6.751(-)	9.933(-)	8.013(-)	9.714(-)

**Table 8:** Median of mean square errors($MME(\hat{\beta })$) and quadratic loss errors($MMR(\hat{D})$) at *τ* = 0.3 for Simulation 3. $MMR(\hat{D})$ are in parentheses.

Sample size	Method	Error distribution			
		*Normal*	*Normalmixtrue*	*Laplace*	*Laplacemixtrue*
n = 30	adGL	0.693(11.078)	0.426(9.632)	0.935(13.41)	0.608(11.588)
	adSpikeGL	**0.207**(**8.668**)	**0.196**(**6.897**)	**0.326**(**9.916**)	**0.236**(**8.558**)
	PMQ	0.916(9.637)	0.757(7.408)	1.352(11.942)	1.012(8.875)
	BL	2.838(-)	2.522(-)	3.815(-)	3.22(-)
	BAL	2.659(-)	2.559(-)	3.799(-)	3.089(-)
	QR	2.977(-)	2.633(-)	3.83(-)	3.15(-)
n = 50	adGL	0.489(8.883)	0.325(7.26)	0.609(10.14)	0.479(8.714)
	adSpikeGL	**0.166**(**5.899**)	**0.13**(**5.429**)	**0.265**(**6.91**)	**0.179**(**5.89**)
	PMQ	0.523(6.773)	0.414(5.967)	0.826(7.842)	0.587(6.097)
	BL	1.971(-)	2.242(-)	2.728(-)	2.757(-)
	BAL	1.899(-)	2.106(-)	2.666(-)	2.663(-)
	QR	2.092(-)	2.444(-)	3.06(-)	2.78(-)
n = 100	adGL	0.261(6.807)	0.15(5.202)	0.394(7.977)	0.254(6.402)
	adSpikeGL	**0.066**(**4.502**)	**0.031**(**3.74**)	**0.182**(**5.552**)	**0.123**(**4.076**)
	PMQ	0.251(5.041)	0.152(3.931)	0.403(5.937)	0.289(4.513)
	BL	1.231(-)	1.557(-)	2.219(-)	2.06(-)
	BAL	1.123(-)	1.454(-)	2.092(-)	1.823(-)
	QR	1.338(-)	1.717(-)	2.297(-)	2.1(-)

**Table 9:** Median of mean square errors($MME(\hat{\beta })$) and quadratic loss errors($MMR(\hat{D})$) at *τ* = 0.5 for Simulation 3. $MMR(\hat{D})$ are in parentheses.

Sample size	Method	Error distribution			
		*Normal*	*Normalmixtrue*	*Laplace*	*Laplacemixtrue*
n = 30	adGL	0.586(11.256)	0.415(9.501)	0.822(12.716)	0.564(11.175)
	adSpikeGL	**0.195**(**7.985**)	**0.172**(**6.351**)	**0.246**(**9.296**)	**0.183**(**8.295**)
	PMQ	0.912(9.032)	0.737(7.37)	0.952(11.751)	0.918(8.996)
	BL	1.733(-)	1.303(-)	1.955(-)	1.401(-)
	BAL	1.729(-)	1.282(-)	1.953(-)	1.445(-)
	QR	2.024(-)	1.479(-)	2.306(-)	1.686(-)
n = 50	adGL	0.428(8.755)	0.309(7.215)	0.453(10.334)	0.394(8.34)
	adSpikeGL	**0.162**(**5.735**)	**0.127**(**5.397**)	**0.164**(**6.863**)	**0.101**(**5.69**)
	PMQ	0.447(6.472)	0.4(5.933)	0.663(7.408)	0.404(5.941)
	BL	0.991(-)	0.886(-)	1.09(-)	0.925(-)
	BAL	0.99(-)	0.899(-)	1.111(-)	0.892(-)
	QR	1.193(-)	1.006(-)	1.314(-)	1.022(-)
n = 100	adGL	0.217(6.604)	0.143(5.139)	0.239(7.822)	0.166(5.982)
	adSpikeGL	**0.068**(**4.448**)	**0.032**(**3.543**)	**0.053**(6.163)	**0.047**(**4.268**)
	PMQ	0.231(4.655)	0.133(3.832)	0.256(**5.194**)	0.169(4.527)
	BL	0.463(-)	0.374(-)	0.53(-)	0.445(-)
	BAL	0.496(-)	0.377(-)	0.53(-)	0.452(-)
	QR	0.523(-)	0.413(-)	0.647(-)	0.481(-)

A few observations can be seen from the results. Firstly, it can be seen from Figs 7–12 that the proposed approaches perform well in general, the adGL generally outperforms the other methods in terms of MAD and RMSE, which indicate explicitly penalizing the off-diagonal covariance components can improve the model selection procedure. Similar findings were also obtained by [32]. On the other hand, the adSpikeGL also has good performance, yet does not perform as well as the adGL. Secondly. Tables 1–9 show that adSpikeGL performs the best in terms of $MME(\hat{\beta })$ and $MMR(\hat{D})$. Take *n* = 50 for an example, adSpikeGL has the smallest $MME(\hat{\beta })$ in 22 out of 36 simulation setups, and the smallest $MMR(\hat{D})$ in 24 out of 36 simulation setups. Though PMQ performs good for *τ* = 0.1, its performance drops when *τ* = 0.3 and *τ* = 0.5, especially in dense case (Simulation 2). Thirdly, Tables 1–9 also display that our approaches work well in terms of MAD, RMSE, $MME(\hat{\beta })$ and $MMR(\hat{D})$ even when the true error term is not ALD, indicating that the LQMMs using ALD in model (2) is merely a working model with artificial assumptions that aim to achieve equivalence between the problems in maximizing ALD and minimizing (3). Finally, as we can observe from Figs 7–12 and Tables 1–9 that, in terms of the four criterions, adGL, adSpikeGl and PMQ all have an overall decreasing trend as the sample size increases, whereas BA, BAL and QR generally fail to estimate accurately, possibly because they almost completely ignore the random effects, which describe within-subject dependence among data, and their performance doesn’t get better as the sample size grows.

For adSpikeGL, the median posterior probability model (MPPM) criteria [33] and the highest posterior probability model (HPPM) criteria are used for model selection, respectively. Tables 10–14 and 11–15 summarize the number of correct and wrong zero coefficients for all cases based on MPPM and HPPM, respectively. On the whole, adSpikeGL generally yield promising results in most cases, even for extreme quantile when *τ* = 0.1 with notoriously challenging. As the sample size grows from 30 to 100, the number of the wrong zero coefficients goes to zero at all considered quantiles, and the number of the correct zero coefficients also become large at moderate and median quantiles. These all indicates good performance of our method in variable selection.

**Table 10:** Average numbers of correct and wrong zero coefficients for fixed effects and random effects in terms of MPPM for Simulation 1. Random effects are in parentheses.

Sample size	*τ*		Error distribution			
			*Normal*	*Normalmixtrue*	*Laplace*	*Laplacemixtrue*
n = 30	p = .1	correct	4.45(4)	4.61(4.77)	4.54(2.15)	4.57(3.71)
		wrong	0.14(0.4)	0.09(0.33)	0.28(0.3)	0.09(0.33)
	p = .3	correct	5.29(4.92)	5.35(5.52)	5.24(3.58)	5.16(4.69)
		wrong	0.12(0.41)	0.09(0.29)	0.2(0.43)	0.09(0.39)
	p = .5	correct	5.29(5.42)	5.37(5.79)	5.27(5.31)	5.45(5.57)
		wrong	0.11(0.45)	0.1(0.43)	0.18(0.73)	0.07(0.49)
n = 50	p = .1	correct	4.44(4.13)	4.53(4.6)	4.47(2.92)	4.63(3.86)
		wrong	0(0.16)	0.01(0.13)	0.03(0.31)	0.02(0.29)
	p = .3	correct	5.48(5.45)	5.38(5.65)	5.25(4.77)	5.23(5.4)
		wrong	0.01(0.21)	0.01(0.13)	0.01(0.4)	0.01(0.35)
	p = .5	correct	5.58(5.78)	5.5(5.85)	5.55(5.68)	5.51(5.87)
		wrong	0(0.35)	0.01(0.18)	0(0.55)	0.01(0.44)
n = 100	p = .1	correct	4.53(3.5)	4.55(3.74)	4.51(2.42)	4.68(2.87)
		wrong	0(0.08)	0(0.01)	0(0.1)	0(0.05)
	p = .3	correct	5.43(5.42)	5.47(5.59)	5.05(5.12)	4.93(5.68)
		wrong	0(0.11)	0(0.02)	0(0.28)	0(0.04)
	p = .5	correct	5.4(5.78)	5.59(5.92)	5.61(5.9)	5.7(5.96)
		wrong	0(0.16)	0(0)	0(0.49)	0(0.13)

**Table 11:** Average numbers of correct and wrong zero coefficients for fixed effects and random effects in terms of HPPM for Simulation 1. Random effects are in parentheses.

Sample size	*τ*		Error distribution			
			*Normal*	*Normalmixtrue*	*Laplace*	*Laplacemixtrue*
n = 30	p = .1	correct	4.43(4.12)	4.61(4.83)	4.5(2.27)	4.53(3.78)
		wrong	0.13(0.38)	0.08(0.35)	0.22(0.32)	0.08(0.35)
	p = .3	correct	5.27(5.07)	5.34(5.52)	5.28(3.8)	5.19(4.8)
		wrong	0.11(0.47)	0.09(0.33)	0.16(0.47)	0.09(0.41)
	p = .5	correct	5.25(5.4)	5.42(5.8)	5.33(5.42)	5.48(5.59)
		wrong	0.11(0.5)	0.11(0.43)	0.17(0.76)	0.07(0.53)
n = 50	p = .1	correct	4.46(4.13)	4.53(4.63)	4.47(2.97)	4.66(3.87)
		wrong	0(0.18)	0.01(0.13)	0.04(0.32)	0.02(0.29)
	p = .3	correct	5.47(5.42)	5.37(5.65)	5.25(4.86)	5.2(5.46)
		wrong	0(0.23)	0.01(0.14)	0.01(0.44)	0.01(0.37)
	p = .5	correct	5.57(5.76)	5.43(5.84)	5.59(5.7)	5.48(5.89)
		wrong	0(0.36)	0.01(0.18)	0(0.59)	0(0.46)
n = 100	p = .1	correct	4.54(3.54)	4.52(3.82)	4.52(2.44)	4.67(2.9)
		wrong	0(0.07)	0(0.01)	0(0.1)	0(0.06)
	p = .3	correct	5.41(5.43)	5.46(5.59)	5.05(5.18)	4.95(5.68)
		wrong	0(0.11)	0(0.01)	0(0.28)	0(0.06)
	p = .5	correct	5.4(5.78)	5.55(5.92)	5.6(5.9)	5.68(5.97)
		wrong	0(0.2)	0(0)	0(0.5)	0(0.13)

**Table 12:** Average numbers of correct and wrong zero coefficients for fixed effects and random effects in terms of MPPM for Simulation 2. Random effects are in parentheses.

Sample size	*τ*		Error distribution			
			*Normal*	*Normalmixtrue*	*Laplace*	*Laplacemixtrue*
n = 30	p = .1	correct	0.11(3.68)	0.25(4.86)	0.01(1.85)	0.21(3.72)
		wrong	2.93(0.27)	1.78(0.29)	3.72(0.29)	2.64(0.31)
	p = .3	correct	0.87(4.27)	0.88(5.46)	0.88(2.32)	0.64(4.54)
		wrong	2.54(0.31)	1.54(0.29)	3.59(0.24)	2.27(0.26)
	p = .5	correct	0.9(4.89)	0.92(5.76)	0.9(4.05)	0.92(5.4)
		wrong	2.27(0.43)	1.48(0.36)	3.07(0.48)	1.99(0.46)
n = 50	p = .1	correct	0.04(3.99)	0.14(4.71)	0.04(2.63)	0.15(3.51)
		wrong	1.45(0.26)	1.07(0.12)	2.33(0.23)	1.55(0.22)
	p = .3	correct	0.87(5.36)	0.86(5.64)	0.61(4.21)	0.49(5.41)
		wrong	1.31(0.29)	1(0.12)	1.88(0.36)	1.31(0.26)
	p = .5	correct	0.93(5.61)	0.94(5.89)	0.93(5.44)	0.94(5.91)
		wrong	1.26(0.35)	0.93(0.18)	1.55(0.52)	1.16(0.39)
n = 100	p = .1	correct	0.01(3.19)	0(3.67)	0(2.25)	0.02(2.93)
		wrong	0.5(0.12)	0.47(0.01)	0.79(0.16)	0.43(0.05)
	p = .3	correct	0.9(5.41)	0.9(5.7)	0.48(5.05)	0.32(5.59)
		wrong	0.5(0.1)	0.48(0.01)	0.61(0.27)	0.35(0.07)
	p = .5	correct	0.93(5.76)	0.95(5.89)	0.93(5.91)	0.91(5.92)
		wrong	0.51(0.22)	0.48(0)	0.55(0.4)	0.3(0.16)

**Table 13:** Average numbers of correct and wrong zero coefficients for fixed effects and random effects in terms of HPPM for Simulation 2. Random effects are in parentheses.

Sample size	*τ*		Error distribution			
			*Normal*	*Normalmixtrue*	*Laplace*	*Laplacemixtrue*
n = 30	p = .1	correct	0.1(3.66)	0.25(4.81)	0.01(1.82)	0.2(3.71)
		wrong	2.97(0.3)	1.64(0.28)	3.65(0.29)	2.59(0.32)
	p = .3	correct	0.89(4.32)	0.88(5.4)	0.83(2.35)	0.66(4.63)
		wrong	2.38(0.34)	1.5(0.3)	3.47(0.26)	2.16(0.34)
	p = .5	correct	0.9(4.95)	0.94(5.78)	0.88(4.01)	0.9(5.46)
		wrong	2.35(0.51)	1.5(0.39)	2.96(0.52)	1.95(0.49)
n = 50	p = .1	correct	0.04(4.03)	0.14(4.72)	0.04(2.65)	0.14(3.56)
		wrong	1.34(0.25)	0.97(0.13)	2.24(0.23)	1.56(0.21)
	p = .3	correct	0.85(5.39)	0.88(5.67)	0.6(4.26)	0.49(5.48)
		wrong	1.26(0.31)	0.89(0.11)	1.79(0.36)	1.28(0.3)
	p = .5	correct	0.93(5.66)	0.93(5.89)	0.93(5.46)	0.93(5.92)
		wrong	1.17(0.37)	0.85(0.18)	1.47(0.57)	1.15(0.4)
n = 100	p = .1	correct	0.01(3.2)	0(3.66)	0(2.31)	0.02(3)
		wrong	0.45(0.12)	0.46(0.01)	0.75(0.16)	0.34(0.06)
	p = .3	correct	0.9(5.36)	0.9(5.7)	0.49(5.11)	0.32(5.6)
		wrong	0.46(0.13)	0.44(0.01)	0.61(0.3)	0.32(0.07)
	p = .5	correct	0.93(5.77)	0.95(5.9)	0.93(5.93)	0.91(5.92)
		wrong	0.44(0.22)	0.44(0)	0.54(0.42)	0.25(0.16)

**Table 14:** Average numbers of correct and wrong zero coefficients for fixed effects and random effects in terms of MPPM for Simulation 3. Random effects are in parentheses.

Sample size	*τ*		Error distribution			
			*Normal*	*Normalmixtrue*	*Laplace*	*Laplacemixtrue*
n = 30	p = .1	correct	6.11(4.04)	6.51(4.91)	6.18(2.5)	6.27(4.09)
		wrong	0(0.43)	0(0.25)	0(0.29)	0(0.43)
	p = .3	correct	6.96(4.8)	7.24(5.5)	7(3.77)	6.97(5.04)
		wrong	0(0.37)	0(0.21)	0(0.39)	0(0.39)
	p = .5	correct	6.99(5.37)	7.32(5.83)	7.13(5.31)	7.2(5.76)
		wrong	0(0.47)	0(0.25)	0(0.66)	0(0.55)
n = 50	p = .1	correct	6.22(4)	6.1(4.73)	6.32(3.05)	6.39(3.83)
		wrong	0(0.21)	0(0.12)	0(0.41)	0(0.27)
	p = .3	correct	7.12(5.32)	7.08(5.73)	6.92(5.07)	6.91(5.51)
		wrong	0(0.22)	0(0.1)	0(0.46)	0(0.31)
	p = .5	correct	7.15(5.63)	7.25(5.88)	7.41(5.83)	7.39(5.95)
		wrong	0(0.38)	0(0.2)	0(0.62)	0(0.37)
n = 100	p = .1	correct	6.36(3.34)	6.47(3.73)	6.49(2.37)	6.54(2.94)
		wrong	0(0.05)	0(0.01)	0(0.16)	0(0.11)
	p = .3	correct	7.27(5.29)	7.42(5.65)	6.95(5.17)	6.96(5.52)
		wrong	0(0.1)	0(0)	0(0.3)	0(0.15)
	p = .5	correct	7.45(5.7)	7.53(5.89)	7.61(5.93)	7.55(5.92)
		wrong	0(0.16)	0(0.04)	0(0.46)	0(0.14)

**Table 15:** Average numbers of correct and wrong zero coefficients for fixed effects and random effects in terms of HPPM for Simulation 3. Random effects are in parentheses.

Sample size	*τ*		Error distribution			
			*Normal*	*Normalmixtrue*	*Laplace*	*Laplacemixtrue*
n = 30	p = .1	correct	6.05(4.11)	6.48(4.94)	6.15(2.69)	6.26(4.14)
		wrong	0(0.43)	0(0.27)	0(0.26)	0(0.39)
	p = .3	correct	6.92(4.84)	7.21(5.58)	6.92(3.94)	6.89(5.07)
		wrong	0(0.37)	0(0.25)	0(0.44)	0(0.42)
	p = .5	correct	6.95(5.46)	7.25(5.83)	7.09(5.39)	7.18(5.83)
		wrong	0(0.51)	0(0.27)	0(0.75)	0(0.59)
n = 50	p = .1	correct	6.2(3.96)	6.24(4.77)	6.26(3.07)	6.38(3.82)
		wrong	0(0.21)	0(0.1)	0(0.37)	0(0.28)
	p = .3	correct	7.08(5.32)	6.99(5.76)	6.92(5.17)	6.88(5.58)
		wrong	0(0.23)	0(0.11)	0(0.51)	0(0.33)
	p = .5	correct	7.13(5.63)	7.25(5.86)	7.4(5.86)	7.38(5.96)
		wrong	0(0.38)	0(0.21)	0(0.66)	0(0.38)
n = 100	p = .1	correct	6.34(3.33)	6.48(3.74)	6.45(2.39)	6.5(2.94)
		wrong	0(0.05)	0(0.01)	0(0.17)	0(0.13)
	p = .3	correct	7.3(5.29)	7.39(5.64)	7.01(5.21)	6.97(5.54)
		wrong	0(0.1)	0(0)	0(0.31)	0(0.15)
	p = .5	correct	7.47(5.72)	7.55(5.88)	7.59(5.96)	7.53(5.92)
		wrong	0(0.17)	0(0.04)	0(0.48)	0(0.15)

Next, in order to evaluate the sensitivity of our methods, we consider the following four different priors of tuning and scale parameters for Simulation 1: case(1) $a_{\lambda_{i}}=b_{\lambda_{i}}=a_{\sigma }=b_{\sigma }=0.1,i=1,2$, case(2) $a_{\lambda_{i}}=b_{\lambda_{i}}=a_{\sigma }=b_{\sigma }=0.01,i=1,2$, case(3) $a_{\lambda_{i}}=b_{\lambda_{i}}=a_{\sigma }=b_{\sigma }=0.001,i=1,2$, case(4) $a_{\lambda_{i}}=b_{\lambda_{i}}=a_{\sigma }=b_{\sigma }=0.0001,i=1,2$. From Table 16, it can be observed that the differences between the four different priors is quite small in general. Hence our methods are not sensitivity to the choice of the hyper-parameters for the priors.

**Table 16:** MAD, RMSE, $MME(\hat{\beta })$ and $MMR(\hat{D})$ for different priors in Simulation 1, when normal mixture error distribution.

Method	*τ*	Prior	MAD	RMSE	$MME(\hat{\beta })$	$MMR(\hat{D})$
adGL	0.1	case (1)	1.957	2.491	1.516	7.439
		case (2)	2.214	2.849	0.839	6.342
		case (3)	2.4	3.073	0.578	6.352
		case (4)	2.439	3.11	0.532	6.632
	0.3	case (1)	1.729	2.239	0.354	7.773
		case (2)	1.795	2.358	0.306	6.353
		case (3)	1.882	2.482	0.265	6.577
		case (4)	1.881	2.513	0.231	6.49
	0.5	case (1)	1.554	1.984	0.324	7.787
		case (2)	1.624	2.124	0.28	6.708
		case (3)	1.685	2.208	0.253	6.799
		case (4)	1.713	2.256	0.259	7.011
adSpikeGL	0.1	case (1)	2.231	2.835	0.987	6.518
		case (2)	2.412	3.058	0.656	6.18
		case (3)	2.461	3.126	0.607	6.389
		case (4)	2.496	3.158	0.498	6.76
	0.3	case (1)	1.84	2.407	0.24	4.762
		case (2)	1.849	2.432	0.24	5.451
		case (3)	1.905	2.494	0.246	5.381
		case (4)	1.946	2.532	0.232	5.765
	0.5	case (1)	1.672	2.212	0.232	5.226
		case (2)	1.741	2.256	0.241	5.996
		case (3)	1.696	2.222	0.24	5.445
		case (4)	1.757	2.263	0.233	5.571

## Real data example

In this section, we use the Age-Related Macular Degeneration Trial (ARMD) data to assess the behavior of the proposed model under different quantiles. This dataset consists of 4 variables, available in the R package **nlme**. Visual acuity of 188 patients with ARMD were measured at 4 time points, resulting in 752 observations. We consider the relationship between the variable of the visual acuity (VISUAL) and three other explanatory variables, including the value of visual acuity measured at baseline(VISUAL0), the time of the measurement(TIME), the treatment indicator(Treat). A detailed description is given in [34]. [34] analyzed the data set using a linear mean mixed model and found that there exist possible interactions between time and treat effects, we then consider the following LQMM (*i* = 1, …, 188, *j* = 1, …, 4):

$$\begin{array}{ccc} VISUAL_{ij} & = & \beta_{0}+\beta_{1}\times VISUAL0_{i}+\beta_{2}\times TIME_{ij}+\beta_{3}\times TREAT_{i}\\ & + & \beta_{4}\times TIME_{ij}\times TREAT_{i}+\alpha_{i0}+\alpha_{i1}\times VISUAL0_{i}\\ & + & \alpha_{i2}\times TIME_{ij}+\alpha_{i3}\times TREAT_{i}\\ & + & \alpha_{i4}\times TIME_{ij}\times TREAT_{i}+\varepsilon_{ij}, \end{array}$$

Here we choose five different values of *τ*, i.e. 0.1, 0.3, 0.5, 0.7, 0.9, to thoroughly describe the response distribution. We implement the adGL, adSpikeGL, PMQ, BL, BAL and QR models on this data set. Table 17 shows the summaries of the MAD and the RMSE, where

$$\begin{array}{c} MAD=(\frac{1}{752}\sum_{i=1}^{188}\sum_{j=1}^{4}\left|y_{ij}-{\hat{y}}_{ij}\right|),\\ RMSE=\sqrt{\frac{1}{752}\sum_{i=1}^{188}\sum_{j=1}^{4}{\left(y_{ij}-{\hat{y}}_{ij}\right)}^{2}}. \end{array}$$

The results in Table 17 suggest that PMQ provides smaller MAD and the RMSE than other methods in upper quantiles(*τ* = 0.7 and *τ* = 09), however, adGL and adSpikeGL perform better in lower and median quantile (*τ* = 0.1, *τ* = 0.3 and *τ* = 0.5). We also report the posterior median estimations and 95% credible intervals for fixed effects and random effect variances in Table, respectively. As in simulations, adGL, adSpikeGL and PMQ are inclined to have similar performance, meanwhile, BAL, BL and QR also are likely to behave similarly, and all methods except for the adSpikeGL do not compress any fixed and random effects to zero.

**Table 17:** MAD and RMSE for the ARMD data.

Criterion	Method	*τ* = 0.1	*τ* = 0.3	*τ* = 0.5	*τ* = 0.7	*τ* = 0.9
MADs	adGL	7.140	5.306	5.078	4.935	6.805
	adSpikeGL	**5.931**	**4.459**	**4.717**	4.970	6.321
	PMQ	7.170	5.959	6.056	**4.251**	**5.871**
	BL	9.138	6.011	5.166	5.936	7.961
	BAL	9.189	6.052	5.211	6.009	7.987
	QR	16.665	10.025	8.712	9.618	13.764
RMSEs	adGL	9.385	7.162	7.256	7.725	9.871
	adSpikeGL	**8.461**	**6.339**	**6.975**	7.726	9.137
	PMQ	9.490	7.820	8.370	**6.870**	**8.909**
	BL	12.786	8.769	8.252	9.739	12.081
	BAL	12.828	8.896	8.351	9.877	12.136
	QR	19.948	12.816	12.120	13.624	17.975

The results of adSpikeGL in Tables 18 and 19 show that (i) for fixed effects, VISUAL0, TIME and TREAT are all identified as effective variables of Visual acuity at all quantiles except for TREAT at extreme quantile (*τ* = 0.1), this may be because treatment has no significant effect on the patients with poor visual acuity, (ii) for random effects, VISUAL0 and TREAT are deemed to be significant predictors of Visual acuity at all quantiles, however, TIME to be insignificant predictor at lower and median quantiles, and (iii) it is worth noticing that the fixed and random effect of the interactions between time and treat effects (TIME×*TREAT*) is not selected by adSpikeGL across all quantiles, suggesting that the effect of the treatment does not change over time no matter what the patient’s vision is.

**Table 18:** Posterior median and 95% credible intervals for the fixed effects of ARMD data.

quantile	Parameters	adGL	adSpikeGL	PMQ	BL	BAL	QR
*τ* = 0.1	*β*_0_	-1.59(-7.493,1.045)	-0.111(-7.424,0.952)	-0.12(-2.21,0.796)	-1.184(-7.773,4.749)	-1.585(-6.614,3.445)	2.897(-)
	*β*_1_	0.896(0.834,1.005)	0.881(0.841,0.999)	0.864(0.831,0.915)	0.915(0.803,1.029)	0.941(0.849,1.027)	0.812(-)
	*β*_2_	-0.21(-0.274,-0.145)	-0.228(-0.282,-0.162)	-0.207(-0.266,-0.139)	-0.222(-0.27,-0.172)	-0.226(-0.274,-0.174)	-0.533(-)
	*β*_3_	-0.426(-3.262,1.11)	0(-3.039,0.749)	-0.125(-1.79,0.695)	-2.492(-6.143,1.073)	-2.443(-5.618,1.039)	-4.374(-)
	*β*_4_	-0.051(-0.147,0.04)	0(-0.119,0.021)	-0.057(-0.156,0.029)	-0.065(-0.138,0.007)	-0.064(-0.136,0.006)	0.031(-)
*τ* = 0.3	*β*_0_	-0.117(-3.049,2.088)	0(-3.025,1.604)	-0.014(-1.191,1.01)	1.552(-3.475,6.39)	1.338(-2.966,5.848)	2.500(-)
	*β*_1_	0.95(0.905,1.004)	0.946(0.913,0.998)	0.943(0.915,0.971)	0.93(0.844,1.013)	0.944(0.868,1.017)	0.900(-)
	*β*_2_	-0.223(-0.285,-0.162)	-0.24(-0.291,-0.177)	-0.224(-0.282,-0.164)	-0.194(-0.245,-0.145)	-0.192(-0.243,-0.144)	-0.265(-)
	*β*_3_	-1.207(-3.612,0.394)	-0.249(-3.319,0.394)	-0.227(-1.866,0.465)	-1.751(-4.568,1.149)	-1.687(-4.404,1.029)	-0.323(-)
	*β*_4_	-0.048(-0.14,0.04)	0(-0.115,0.018)	-0.052(-0.142,0.036)	-0.061(-0.129,0.006)	-0.066(-0.133,0.002)	-0.126(-)
*τ* = 0.5	*β*_0_	0.975(-0.965,5.282)	0.004(-0.935,4.763)	0.171(-0.931,2.569)	4.185(0.052,8.845)	3.765(-0.251,8.174)	2.839(-)
	*β*_1_	0.975(0.901,1.018)	0.983(0.909,1.014)	0.986(0.945,1.013)	0.926(0.847,0.994)	0.937(0.866,1.001)	0.946(-)
	*β*_2_	-0.197(-0.256,-0.136)	-0.219(-0.268,-0.157)	-0.197(-0.255,-0.14)	-0.143(-0.185,-0.104)	-0.142(-0.185,-0.102)	-0.151(-)
	*β*_3_	-0.872(-3.133,0.507)	-0.072(-2.622,0.507)	-0.235(-1.903,0.615)	-1.391(-3.917,1.045)	-1.204(-3.729,1.169)	-0.656(-)
	*β*_4_	-0.058(-0.147,0.028)	0(-0.118,0.012)	-0.057(-0.145,0.026)	-0.073(-0.131,-0.015)	-0.073(-0.132,-0.013)	-0.075(-)
*τ* = 0.7	*β*_0_	6.253(1.701,10.526)	4.644(-0.128,10.815)	3.118(-0.276,8.154)	6.422(1.923,11.117)	5.304(1.124,9.614)	6.749(-)
	*β*_1_	0.929(0.855,1.008)	0.949(0.852,1.032)	0.977(0.897,1.036)	0.924(0.847,0.997)	0.942(0.873,1.008)	0.929(-)
	*β*_2_	-0.176(-0.236,-0.118)	-0.197(-0.247,-0.135)	-0.181(-0.241,-0.122)	-0.11(-0.145,-0.075)	-0.109(-0.143,-0.074)	-0.074(-)
	*β*_3_	-1.09(-3.435,0.401)	-0.045(-2.521,0.616)	-0.52(-2.538,0.964)	-1.155(-3.589,1.19)	-0.891(-3.226,1.363)	-1.451(-)
	*β*_4_	-0.056(-0.14,0.029)	0(-0.122,0.012)	-0.05(-0.139,0.036)	-0.063(-0.115,-0.01)	-0.06(-0.115,-0.007)	-0.063(-)
*τ* = 0.9	*β*_0_	12.515(7.598,18.005)	9.273(3.135,14.157)	7.479(2.442,12.155)	8.299(3.633,13.256)	6.382(2.174,10.956)	17.773(-)
	*β*_1_	0.87(0.775,0.952)	0.912(0.834,1.007)	0.94(0.87,1.021)	0.938(0.858,1.015)	0.966(0.89,1.033)	0.818(-)
	*β*_2_	-0.174(-0.237,-0.108)	-0.189(-0.238,-0.128)	-0.18(-0.235,-0.121)	-0.091(-0.124,-0.058)	-0.089(-0.123,-0.051)	-0.011(-)
	*β*_3_	-2.257(-5.291,0.092)	-0.252(-3.101,0.51)	-0.576(-2.655,1.234)	-1.087(-3.599,1.17)	-0.873(-2.955,1.401)	-2.636(-)
	*β*_4_	-0.043(-0.134,0.045)	0(-0.117,0.016)	-0.041(-0.12,0.048)	-0.051(-0.102,0.003)	-0.048(-0.099,0.007)	0.023(-)

**Table 19:** Posterior median and 95% credible intervals for the random effect variances of ARMD data.

*τ*	Parameters	adGL	adSpikeGL	PMQ
*τ* = 0.1	*σ*_00_	3.182(0.016,14.82)	91.361(62.646,130.536)	0.251(0,22.157)
	*σ*_11_	0.023(0.007,0.035)	0(0,0)	0.029(0.01,0.043)
	*σ*_22_	0.076(0.051,0.109)	0.077(0.058,0.102)	0.073(0.05,0.106)
	*σ*_33_	1.236(0.031,8.159)	247.861(113.119,422.548)	1.249(0.002,85.935)
	*σ*_44_	0.025(0.001,0.121)	0(0,0)	0.065(0,0.253)
*τ* = 0.3	*σ*_00_	0.981(0.002,8.525)	30.736(15.537,56.308)	0.087(0,12.763)
	*σ*_11_	0.008(0.002,0.014)	0(0,0.003)	0.007(0.001,0.013)
	*σ*_22_	0.072(0.049,0.103)	0.072(0.053,0.096)	0.072(0.049,0.102)
	*σ*_33_	4.622(0.218,13.325)	137.31(35.006,217.77)	20.295(0.957,144.869)
	*σ*_44_	0.026(0.002,0.104)	0(0,0)	0.007(0,0.102)
*τ* = 0.5	*σ*_00_	0.591(0,10.282)	27.488(15.813,46.503)	1.387(0,51.397)
	*σ*_11_	0.007(0.001,0.014)	0(0,0)	0.004(0,0.011)
	*σ*_22_	0.068(0.046,0.104)	0.066(0.048,0.09)	0.067(0.047,0.098)
	*σ*_33_	3.363(0.1,10.958)	81.255(2.149,172.215)	14.283(0.082,97.629)
	*σ*_44_	0.017(0.001,0.153)	0(0,0)	0.006(0,0.114)
*τ* = 0.7	*σ*_00_	10.218(2.272,24.638)	143.485(78.765,280.103)	90.912(28.974,222.612)
	*σ*_11_	0.002(0,0.007)	0.013(0.004,0.048)	0.005(0,0.028)
	*σ*_22_	0.062(0.04,0.088)	0.063(0.045,0.087)	0.064(0.042,0.094)
	*σ*_33_	1.795(0.045,9.234)	54.378(4.052,162.822)	2.581(0.005,80.103)
	*σ*_44_	0.016(0.001,0.093)	0(0,0)	0.006(0,0.115)
*τ* = 0.9	*σ*_00_	21.142(0.001,40.1)	162.243(104.606,339.58)	170.374(92.769,301.983)
	*σ*_11_	0.002(0,0.03)	0.013(0.004,0.067)	0.013(0.003,0.036)
	*σ*_22_	0.065(0.039,0.379)	0.066(0.048,0.089)	0.074(0.048,0.109)
	*σ*_33_	1.168(0.026,8.957)	50.279(5.654,124.126)	2.636(0.004,74.622)
	*σ*_44_	0.017(0.001,0.389)	0(0,0)	0.028(0,0.199)

## Conclusion and discussion

In this work, we presented a novel shrinkage method to linear quantile mixed model for analysing and interpreting longitudinal data. We also consider the variable selection based on a Bayesian adaptive lasso and an extended Bayesian adaptive group lasso with spike and slab priors. Unlike other approaches for LQMMs, which need some ad hoc methods to do model selection, adSpikeGL can directly identify the significant fixed and random effects simultaneously. Several criterions were adopted to evaluate the finite sample performance of the proposed methods thoroughly by simulations and real dat example. The results reveal that the proposed methods is very competitive with the existing methods. On the other hand, as the sample size gets large, adGL and adGLSpike generally tend to provide smaller MMAD, MRMSE, $MME(\hat{\beta })$ and $MMR(\hat{D})$ across all scenarios, and adGLSpike also has good performance in variable selection in most cases. The only exception is at *τ* = 0.1 where the number of the correct zero coefficients of adGLSpike did not show an increasing trend with sample size. The problem can be caused by the drawbacks of the ALD that do not allow the skewness, kurtosis and tails of distribution to vary. To overcome this problem [35] recently introduced a generalized asymmetric Laplace distribution (GALD) to establish QR in linear regression. In the future, we would like to extend the GALD to the linear mixed effect models. Moreover, we would also like to consider some other Bayesian shrinkage priors, such as the double-Pareto prior [36], horseshoe prior [37], the normal-gamma prior [38], etc, though the lasso-type priors is the most widely used shrinkage priors in the literature.

## Supporting information

S1 File (RAR)
